# Indole-Acetic Acid Promotes Ammonia Removal Through Heterotrophic Nitrification, Aerobic Denitrification With Mixed *Enterobacter* sp. Z1 and *Klebsiella* sp. Z2

**DOI:** 10.3389/fmicb.2022.929036

**Published:** 2022-07-08

**Authors:** Yuxiao Zhang, Qing Xu, Gejiao Wang, Kaixiang Shi

**Affiliations:** State Key Laboratory of Agricultural Microbiology, College of Life Science and Technology, Huazhong Agricultural University, Wuhan, China

**Keywords:** *Enterobacter* sp., *Klebsiella* sp., heterotrophic nitrification–aerobic denitrification, indole-acetic acid, ammonia removal

## Abstract

Mixed *Enterobacter* sp. Z1 and *Klebsiella* sp. Z2 displayed an outstanding ammonia removal capacity than using a single strain. Metabolomics, proteomics, and RNA interference analysis demonstrated that the HNAD process was closely related to indole-acetic acid (IAA). Under the cocultured conditions, the excess IAA produced by Z2 could be absorbed by Z1 to compensate for the deficiency of IAA in the cells. IAA directly induced the expression of denitrifying enzymes and further activated the IAA metabolism level, thus greatly improving the nitrogen removal ability of Z1. In turn, nitrate and nitrite induced the expression of key enzymes in the IAA pathways. Moreover, Z1 and Z2 enhanced two IAA metabolic pathways in the process of mixed removal process. The activated hydrolysis-redox pathway in Z1 reduced the oxidative stress level, and the activated decarboxylation pathway in Z2 promoted intracellular energy metabolism, which indirectly promoted the process of HNAD in the system.

**Figure:**
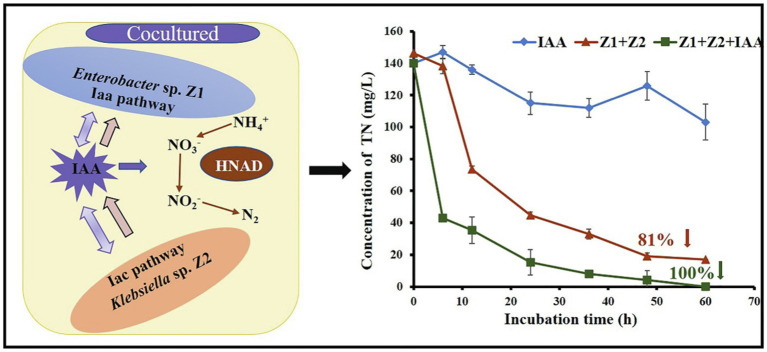
Graphical Abstract

## Highlights

– Indole-acetic acid (IAA) accumulated during bacterial cocultured nitrogen removal.
– IAA enhanced denitrification gene expression and nitrogen removal efficiency.
– The hydrolysis-redox pathway in Z1 is activated and promotes nitrogen removal.
– The decarboxylation pathway in Z2 is used to promote energy metabolism.

## Introduction

Due to the high accumulation of nitrogen in water, soil, atmosphere, and organisms, severe eutrophication and water quality deterioration has occurred, while nitrite, a common form of inorganic nitrogen, proved toxic to aquatic organisms and humans (Jensen, 2003; Zhou et al., 2007; Galloway et al., 2008; Ma et al., 2022). Therefore, nitrogen treatment has become a focus of widespread attention. Microorganisms have been widely used in the study of nitrogen removal because of their high efficiency, strong stability, and low secondary pollution (Priji et al., 2013, 2016; Chen et al., 2021; Supplementary Table S1). Traditional biostrategies have been extensively explored and applied to actual nitrogen-contaminated wastewater, but there are generally bottlenecks and obstacles of low efficiency and poor stability. In recent years, heterotrophic nitrification and aerobic denitrification (HNAD), as a continuous nitrogen removal pathway, has been widely reported. To date, many efficient strains have been reported to convert ammonia, nitrate, and nitrite to nitrogen gas (Li et al., 2016; Zhang et al., 2018; Song et al., 2021).

To explore the mechanisms affecting the nitrogen removal capacity of HNAD bacteria, some previous studies have been conducted (Supplementary Table S2). A study conducted in 2016 showed that fulvic acid could improve the total nitrogen removal rate and reduce nitrite accumulation by *Paracoccus denitrificans* with electron transport (Li et al., 2016). Zhang et al. (2018) found that the generation of biofilms is conducive to maintaining the stability of the nitrogen removal process in *P. denitrificans*. Sun et al. (2016) revealed that the presence of heavy metals inhibited the nitrogen removal capacity of *Cupriavidus* sp. S1. In addition, the degradation of active aromatic hydrocarbons has been reported to be mutually coupled with the HNAD process (Zhu et al., 2020). While the above reports have revealed some influencing factors, the regulatory mechanism of cell growth and metabolism in the HNAD process remains unclear. In addition, previous reports have verified the nitrogen removal ability of HNAD strains in detail under laboratory conditions, but research on nitrogen removal capacity and multicycle stability in natural wastewater is also limited.

Indole-acetic acid (IAA), a common plant hormone, is involved mainly in the division, extension, and differentiation of plant cells, as well as other developmental processes (Fu and Wang, 2011; Donati et al., 2013). Many species of bacteria can produce and secrete IAA in the process of their growth (Sergeeva et al., 2002). IAA can pass through sources of carbon and nitrogen so that many bacteria can also degrade IAA through hydrolysis-redox and decarboxylation, respectively (Mino, 1970; Leveau and Lindow, 2005; Scott et al., 2013; Duca et al., 2014). Bacterial hydrolysis-redox of IAA occurs around the secondary metabolite anthranilic acid under the action of the *iaa* cluster, and decarboxylation is the pathway under the action of the *iac* cluster with catechol as the key intermediate metabolite (Duca et al., 2014). IAA can induce bacteria to better adapt to stressful environmental conditions, thus endowing strains with greater evolutionary adaptability (Spaepen et al., 2007; Kim et al., 2011). Nitrite, a potentially toxic substance accumulated during the nitrogen removal process in the HNAD strains, increased their oxidation pressure (Li et al., 2016; Zhang et al., 2019). However, the regulation of IAA of the intracellular stress response, energy flow, and other metabolic processes in HNAD strains remains unclear.

In our previous study, *Enterobacter* sp. Z1 and *Klebsiella* sp. Z2 were confirmed to be HNAD bacteria (Zhang et al., 2019). In comparison to the single strain, the combined use of strains Z1 and Z2 showed a higher capacity for ammonia removal, and the amount of nitrogen decreased to the first level standard of surface water after removal (Zhang et al., 2019). However, the interaction between the HNAD bacteria Z1 and Z2 has not yet been determined. In this study, metabolomics, proteomics, quantitative real-time polymerase chain reaction (RT-qPCR), and RNA interference were performed to clarify the intercellular complement of IAA metabolism in strains Z1 and Z2, which promotes simultaneous processes of HNAD in mixed microbial agents. This study provides an extensive theoretical basis for the future application of bioremediation of water eutrophication.

## Materials and Methods

### Strains, Media, and Primers

*Enterobacter* sp. Z1 (SSWR00000000) and *Klebsiella* sp. Z2 (SSWS00000000) were used in this study, and strains Z1 and Z2 were cultured in the BM (ammonium/succinate), NM (ammonium/acetate) or DM (nitrate or nitrite, acetate) media as described in Zhang et al. (2019). Moreover, strains Z1 and Z2 were cultured in NM, DM or BM media with 0.5% initial inoculation at 28°C and 150 rpm in this study. BM contained 0.47 g of (NH_4_)_2_SO_4_, 5.49 g of sodium succinate, 0.2 g of MgSO_4_∙7H_2_O, 0.5 g of NaH_2_PO_4_∙H_2_O, 0.1 g of CaCl_2_∙2H_2_O, and 0.5 g of K_2_HPO_4_ per liter at pH 7.2. NM comprised 10 g of CH_3_COONa, 7.0 g of K_2_HPO_4_, 3.0 g of KH_2_PO_4_, 0.1 g of MgSO_4_·7H_2_O, 0.05 g of FeSO_4_·7H_2_O, 100 mg of (NH_4_)_2_SO_4_ and pH 7.2. DM included 10 g of CH_3_COONa, 7.0 g of K_2_HPO_4_, 3.0 g of KH_2_PO_4_, 0.1 g of MgSO_4_·7H_2_O, 0.05 g of FeSO_4_·7H_2_O, 100 mg KNO_3_ (DM-1) or 100 mg NaNO_2_ (DM-2) per liter at pH 7.2. The agar plates contained 18 g agar per liter, and all media were autoclaved at 121°C for 15 min. Basal medium (BM) was used for bacterial enrichment and isolation, and Nitrification Medium (NM) and Denitrification Medium (BM) were used to determine the nitrification and denitrification ability (Padhi et al., 2013; He et al., 2016). The specific medium composition and primers used in this study are listed in the Supplementary Material.

### Metabolomics and Proteomics Analysis

Cells of strain Z1 and strain Z1 + Z2 were cultured in NM medium for 48 h, and the supernatant was collected as extracellular metabolites by centrifugation (8,000 rpm, 4°C, 10 min). Then, metabolomics analysis was performed by Majorbio Inc. (Shanghai, China; Wang et al., 2020). Extracellular active metabolites in the LB culture medium were analyzed by metabolomics. After mixing the filtered 100 μl sterile supernatant with 400 μl precooled methanol, the metabolites were extracted according to the instructions (Majorbio Bio-Pharm Technology Co., Shanghai, China), and the extracted samples were detected by an AB SCIEX liquid chromatography/mass spectrometry (LC/MS) system (UPLC-TripleTof).

Strains Z1 and Z2 were cultured in DM medium for 36 h under the conditions of a single culture and coculture with or without the addition of 10 mg/l IAA, and then the cell pellet was collected by centrifugation (12,000 rpm, 4°C) and freeze-dried. Then, 200 μl of L3 buffer (ingredient by Gene-create Company) and 800 μl precooled acetone (containing 10 mM dithiothreitol) were added. Next, the collected sediment was centrifuged at 12,000 rpm for 20 min at 4°C. The steps described above were repeated one more time, and the sediment was dissolved in 100 μl L3 buffer. The concentration of protein was measured by the Bradford method (Martinez-Esteso et al., 2014).

Proteins (100 mg) from each sample were digested with 0.5 ml NH_4_HCO3 (50 mM) and 2 μg Trypsin for 12–16 h at 37°C. Then, the samples were acidified with an equal amount of 0.1% formic acid added to trypsin peptide and the chromatoid-xc18 column three times. The strata-x C18 column was activated with 1 ml methanol and balanced with 1 ml 0.1% formic acid. The column was then cleaned twice with 0.1% formic acid and 5% acetonitrile, and finally, 1 ml of 0.1% formic acid and 80% acetonitrile were added to collect the solution and vacuum-dried. About 0.5 TEAB 20 μl was used to dilute the dry powder peptide, and the sample was labeled 8-plex (113, 114, 115, 116, 117, 118, 119, and 120 Da; Martinez-Esteso et al., 2014).

The mass spectrometer was AB SCIEX nanolc-ms/MS (Triple TOF 5600 plus). The analytical column was an AB SCIEX column (within a radius of 75 μM, fill 3 μM, 120 ChromXP C18, 10 cm length). The nozzle is a new target (within a radius of 20 μM, diameter of 10 μM). The capture column was an eksigent Chromxp trap column (3 μm C18-CL, 120 Å, 350 μm × 0.5 mm). Data processing was conducted using Proteinpilot^™^V4.0.8085, and the comparison of the *Enterobacter* sp. Z1 was conducted by search. Proteinpilots were set to the following values: type search (iTRAQ 8 plexus), enzyme (trypsin), cysteine alkylation (iodoacetamide), instrument (TripleTOF 5,600), deviation correction (true), background correction (true), ID focus (biological modification), search (complete ID), protein quality (unlimited), and database (UniProt root cancer soil bacillus). The differentially expressed proteins were classified using three databases: WebMD, National Center for Biotechnology Information (NCBI), and Kyoto Encyclopedia of Genes and Genomes (KEGG).

### Determination of IAA, DO, COD Content, and Nitrogen Removal Rate

For a single strain, 500 μl suspension (OD_600_ = 1.0) was inoculated in 100 ml NM, DM, or BM at 28°C to reach the initial inoculation amount of 0.5%, then shaken at 150 rpm. Under the cocultured conditions, 250 μl suspensions of strains Z1 and Z2 (OD_600_ = 1.0) were added. The supernatant samples were collected every 6 h to detect the concentration of IAA. Two milliliters of Salkowski reagent (1.7% FeCl_3_ in 37% H_2_SO_4_) were added to 1 ml of the above supernatant. The absorbance was measured at 540 nm after incubation at 37°C for 30 min and then compared with the standard curve (Glickmann and Dessaux, 1995; Lin et al., 2018). Meanwhile, the concentration of DO and COD was determined by Shao et al. (2020).

Meanwhile, the different nitrogen removal abilities of the strain after adding exogenous IAA were detected. Strains Z1 and Z2 were inoculated with or without 10 mg/l IAA according to the above inoculation amount. Samples were taken every 6 h to determine the concentration of ammonia, nitrate, nitrite and total nitrogen (Zhang et al., 2019, 2020). Potassium persulfate spectrophotometry was used for total nitrogen, and Nessler spectrophotometry was used for ammonia. Nitrate and nitrite were measured by He et al. (2016) and Wang et al. (2013).

### Real-Time Quantitative PCR

In this study, Real-time quantitative PCR (RT-qPCR) was used to detect the expression levels of key genes associated with HNAD and IAA metabolic processes. Strains Z1 and Z2 were cultured in NM, DM or BM medium with 0.5% initial inoculation at 28°C and 150 rpm for 36 h, and the cell precipitates were collected for RT-qPCR analysis. Total RNA was extracted with TRIzol (Invitrogen), genomic DNA was removed with RNase-free Dnase I (Takara) enzyme digestion at 37°C, and 50 mM EDTA was finally added. The enzymatic hydrolysis was terminated by thermal stimulation at 65°C for 10 min, and cDNA was synthesized using a reverse transcriptase kit (Fermentas). After the RNA concentration was determined spectrophotometrically (NanoDrop 2000, Thermo), 300 ng of total RNA was reduced to cDNA using the Revert Aid First Strand cDNA Synthesis Kit (Thermo). Finally, the cDNA was diluted 10-fold and analyzed by RT-qPCR with SYBR Green Real-time PCR Master Mix (Toyobo).

Primers were designed to detect the genetic sequence of the proteins (single copy of each protein), and the results were analyzed using 2^−ΔΔCT^ with an iQ5 real-time PCR detection system (American—Bio-Rad; Wang et al., 2017). Quantitative RT-qPCR was performed on a 0.1 ml fast optical 96-well reaction plate (ABI) in an ABI VIIA7.

### RNA Interference

Due to the extensive antibiotic resistance of strains Z1 and Z2, RNA interference (RNAi) technology was used to add strong promoter TacP (Kagiya et al., 2005) and silencing fragments of targeted mRNA sequence-specific gene into the plasmid pUC19, which was labeled with a red fluorescent protein (Han, 2018). Target genes, *iaaA* and *iacA* in strains Z1 and Z2, were identified for RNAi, and primers for amplifying target genes are listed in Supplementary Table S3. The target gene was inserted into *lacZ* gene of the plasmid by *Hin*d III and *Sac* I, and the recombinant plasmid was transferred into strains Z1 and Z2 by electroporation. Finally, the wild strains without plasmid insertion showed white colonies on the LB plate without red fluorescence, the strains with blank plasmid insertion showed red fluorescence and blue colonies on the LB plate, while the RNAi strains with target fragment insertion showed white colonies with red fluorescence.

Meanwhile, samples were taken every 12 h, and RT-qPCR was used to detect the expression level of silenced genes in RNAi strains, which showed a stable gene-silencing effect. The results of phenotypic verification and gene interference stability test of RNAi strains are shown in Supplementary Figure S3.

### H_2_O_2_ and Oxidative Stress Detection

In this study, we determined the concentration of H_2_O_2_ with the spectrofluorometry method. After being cultured for 24 h, bacterial cells were collected (centrifuged at 12,000 rpm and 4°C for 5 min) and washed twice with 50 mmol/l K_3_PO_4_ (pH 7.8). After 5 min of ultrasonic crushing of cells on ice, the supernatant was taken, and the content of H_2_O_2_ in cells was determined by the method of Li et al. (2017).

In addition, we examined the effects of IAA metabolism on oxidative stress tolerance by wild-type and RNA-interfering strains. Five hundred microliters of bacterial suspension were added to a 96-microtitration plate after the bacteria were cultured to the mid-log stage (OD600 = 0.6) and treated with 5 mM H_2_O_2_ for 20 min (Herigstad et al., 2001; He et al., 2018). The survival rate of different treatment groups was determined by dilution plate counting before and after treatment (Zhang et al., 2019).

### Determination of the Amounts of Adenosine Triphosphate and Nicotine Adenine Dinucleotide Hydride

The cells were collected and cultured for 12 and 36 h by centrifugation at 4°C at 5,000 rpm for 5 min and then resuspended in a mixture of 1 ml perchloric acid (0.4 M) and 200 μl ethylenediaminetetraacetic acid (EDTA, 1 mM). After ultrasonic treatment on ice for 5 min, the cells were centrifuged at 12,000 rpm for 5 min at 4°C and filtered through a 0.22-μm filtration membrane. In this study, the concentrations of Adenosine triphosphate (ATP) and Nicotine adenine dinucleotide hydride (NADH) in cells were analyzed by high-performance liquid chromatography (HPLC; HPLC 2690 Series, Waters, Massachusetts). The mobile phase contained 90% 50 mM phosphate buffer containing 10% acetonitrile and 3.22 g/l tetrabutylammonium bromide (pH 6.8), and the flow rate was 1 ml/min (Wang et al., 2017; Zhang et al., 2019).

### Data Analysis

In this study, Excel 2013 was used for data analysis without special annotation, and the results are expressed as the mean ± SD (standard deviation). The removal rate is calculated as (C_0_ − C_t_)/t, and the removal percentage is calculated as (C_0_ − C_t_)/C_0_*100%, where C_0_ is the initial concentration and C_t_ is the final concentration at time t. Sequential growth efficiency (%) is calculated as (C_2_ − C_1_)/C_1_, where C_1_ is the removal rate of the strain without IAA, and C_2_ is the removal rate of the strain with the addition of IAA.

## Results

### Metabolomics Analysis Revealed Metabolites Associated With Ammonia Removal in Mixed Strains

**Figure 1:**
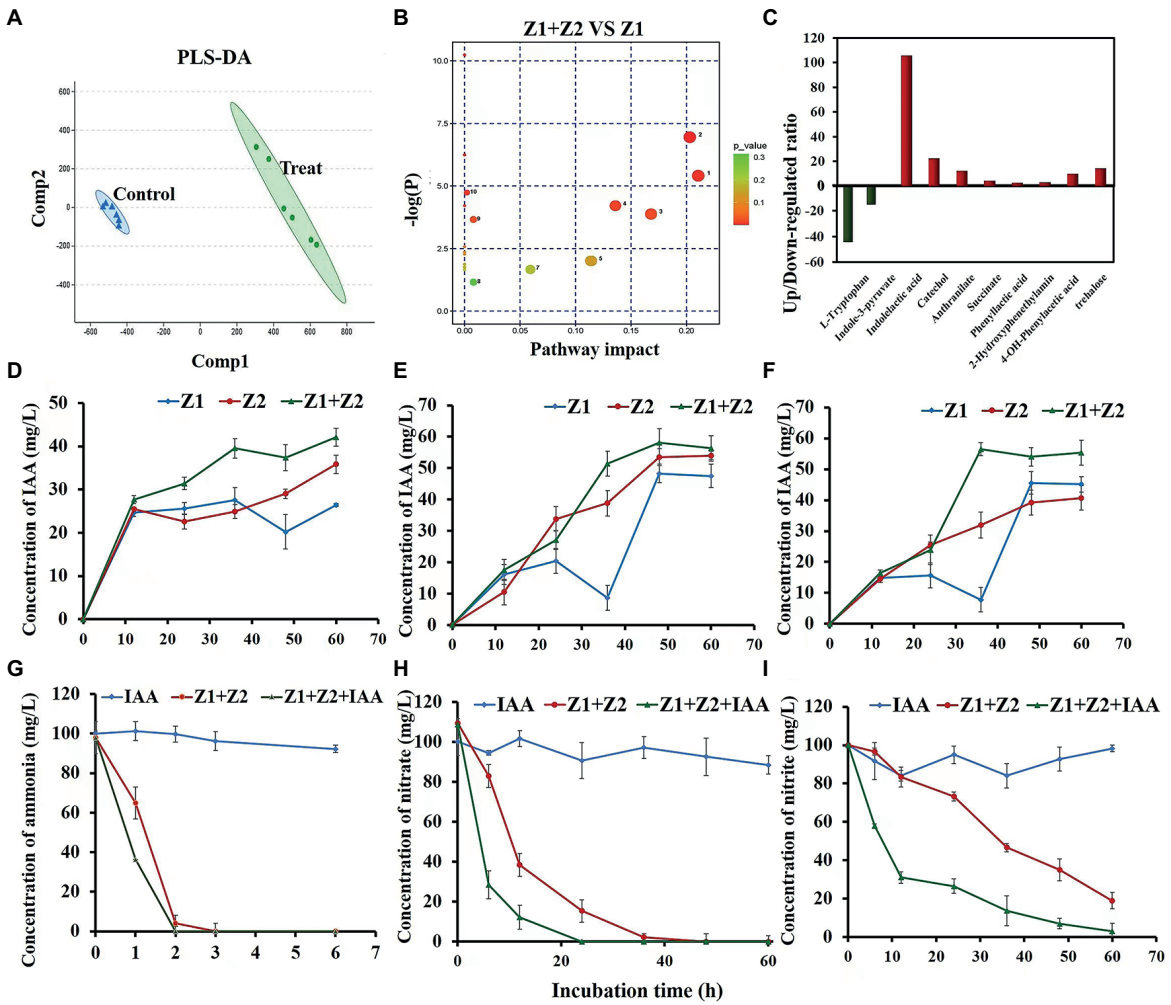
Cellular metabolomics analysis and phenotypic validation of strains Z1 and Z2 in response to ammonia and IAA exposure. **(A)** Represents PLS-DA of metabolite spectrum by LC/MS. Points of the same color represent six repetitions in the same treatment group. **(B)** Represents pathway impact analysis of strain Z1 of a single culture and cocultured conditions in the presence of NH_4_^+^. The map was generated using MetaboAnalyst 4.0 and the KEGG metabolic network, and the pathways with impact value ≥ 0.10 were numbered. Significance ranges from moderate (yellow) to highly significant (red), and the size of the circles correlated with the number of metabolites identified by LC/MS analysis for each pathway: 1, tryptophan metabolism; 2, lysine degradation; 3, phenylalanine metabolism; 4, phenylalanine, tyrosine, and tryptophan biosynthesis; 5, biosynthesis of plant hormones; 6, lysine biosynthesis; 7, purine metabolism; 8, sphingolipid metabolism; 9, glucosinolate biosynthesis; and 10, indole alkaloid biosynthesis. **(C)** Represents the upregulated/downregulated ratio of key substances in IAA synthesis and metabolism. **(D–F)** Represent the concentrations of IAA during the removal process of ammonia, nitrate, and nitrite, respectively. **(G–I)** Represent the removal ability of ammonia, nitrate, and nitrite with or without 10 mg/l IAA. Values represent the mean ± standard deviation of three replicates.

In a previous study, we found that the ammonia removal process in the system was greatly accelerated after the coculture of strains Z1 and Z2 (Zhang et al., 2019). To explore the mechanism, metabolomics analysis was performed in this study, and partial least squares—discriminant analysis (PLS-DA) was executed to intuitively observe the overall metabolic differences between monocultures and cocultures during ammonia removal (Figure 1A). The results showed significant metabolic differences between the two systems, with 104 metabolites significantly regulated upward and 15 metabolites significantly regulated downward. Metabolic pathways of the metabolites were analyzed with significant differences between the synthesis and metabolism of aromatic amino acids, the biosynthesis of plant hormones, and the metabolism of sphingolipids (Figure 1B). Interestingly, further analysis of metabolomics results indicated that IAA and its secondary metabolites catechol and anthranilate were upregulated by 105, 22.4, and 12.2 times, respectively, while the synthetic substrate of IAA, L-tryptophan, was significantly downregulated by 44 times.

### The Addition of IAA Increased HNAD Capacity Using Mixed Strains

Phenotypic experiments were then checked for IAA accumulation in the mixed cells during ammonia, nitrate, and nitrite removal processes. Compared with the single strain conditions, the amount of IAA in the mixed strains was significantly increased under ammonia, nitrate, and nitrite conditions, reaching 42.1, 56.3, and 55.3 mg/l, respectively (Figures 1D–F). In the process of removal of nitrates and nitrites by strain Z1, the accumulation of IAA decreased in the intermediate stage (~36 h), then gradually increased (Figures 1E,F).

An additional 10 mg/l exogenous IAA was used to explore the direct effect of IAA on the HNAD process. In both monocultured and cocultured systems, the addition of exogenous IAA improved the rate of nitrogen removal. Under the cocultured condition, the removal rates of ammonia, nitrate, and nitrite increased by 27.5%, 60.7%, and 23.1%, respectively, with the addition of IAA (Figures 1G–I). The above results showed that the addition of exogenous IAA had the maximal effect on nitrate removal, which was completely removed in 24 h (Figure 1H). In addition, after adding exogenous IAA, the mixed strains could completely remove nitrite in the system within 60 h without any accumulation (Figure 1I).

In the process of HNAD, the content of DO and COD were detected synchronously (Supplementary Table S4), and the results showed that DO and COD content in different systems did not change significantly, and remained about 8–9 mg/l and 50–60 mg/l, respectively.

### Proteomics Revealed Two Major Metabolic Pathways of IAA in Strains Z1 and Z2

#### Comparative Proteomics Assays

**Figure 2:**
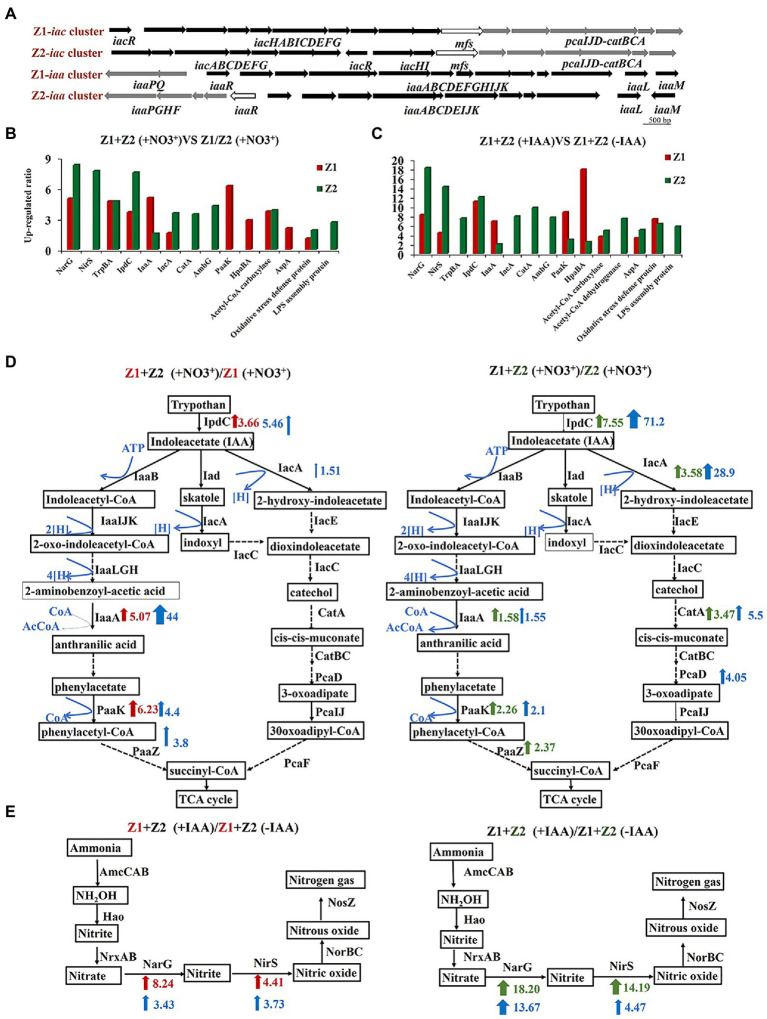
Genomic and iTRAQ proteomics analysis of cellular metabolism of strains Z1 and Z2 in response to nitrate and IAA exposure. **(A)** Represents gene clusters of Iaa and Iac, key enzymes of IAA metabolism, in strains Z1 and Z2. The map was generated using NCBI and KEGG metabolic network. **(B)** Represents the key differentially expressed proteins in the cocultured conditions compared with the pure cultured conditions in the presence of NO_3_^−^. Red columns represent the proteins from strain Z1 and green columns represent the proteins from strain Z2. **(C)** Represents the key differentially expressed proteins in cocultured conditions with or without the addition of IAA. **(D)** Represents the differentially expressed proteins in IAA pathways in strains Z1 (left) or Z2 (right) in cocultured conditions compared with the pure cultured conditions. **(E)** Represents the differentially expressed proteins in the ammonia removal process with the addition of IAA compared with the absence of IAA. Red bar arrows represent upregulated proteins, and green bars and arrows represent downregulated proteins. Blue range arrows suggest upregulated genes encoding the corresponding proteins based on RT-qPCR.

The results of the genome-level analysis showed that strains Z1 and Z2 can synthesize IAA through the indole-3-pyruvate (IPA) pathway, and both can degrade IAA through hydrolyzation-redox (*iaa* pathway) or decarboxylation (*iac* pathway). The analysis of *iaa* and *iac* gene clusters in strains Z1 and Z2 indicated that the two gene clusters were generally conserved, and there were some differences in the location and transcription direction of regulatory genes (Figure 2A).

To explain why IAA accumulation increased in mixed strains, we performed iTRAQ proteomics in two groups: (1) monocultured and cocultured conditions, and (2) cocultured conditions with or without 10 mg/l IAA. Compared with the single HNAD process, 48 proteins were significantly upregulated, and 60 proteins were downregulated in strain Z1 under the cocultured conditions (Supplementary Figure S1A). In strain Z2 under the same conditions, 64 proteins were upregulated, and 86 proteins were downregulated (Supplementary Figure S1B). Compared with the cocultured conditions without 10 mg/l exogenous IAA, 53 proteins of strain Z1 were significantly upregulated and 39 proteins were significantly downregulated (Supplementary Figure S1C). In strain Z2, 92 proteins were significantly upregulated, and 85 proteins were significantly downregulated (Supplementary Figure S1D). The primary metabolic pathways involved denitrification, nitrogen removal, tryptophan metabolism, indole-acetic acid metabolism, phenylalanine metabolism, extracellular polysaccharide, oxidative stress, and energy metabolism (Supplementary Figures S1E–H).

#### Two Major Metabolic Pathways of IAA in Strains Z1 and Z2

To study the differences in IAA metabolism pathways in monocultured and cocultured conditions, we focused on the expression of related proteins in different IAA systems. Compared with the single HNAD process, the key enzymes of IAA synthesis, indolepyruvate decarboxylase IpdC, in strains Z1 and Z2, were upregulated by 3.66 and 7.55 times, respectively, and the key enzymes, 3-oxoadipyl-CoA thiolase IaaA and phenylacetate-CoA oxygenase PaaK, in hydrolysis-redox metabolism were upregulated by 5.07, 1.58 and 6.23, 2.26 times, respectively (Figures 2B,C). Meanwhile, acyl-CoA dehydrogenase IacA, a key protein in the decarboxylation pathway, was upregulated by 1.51- and 3.58-fold in strains Z1 and Z2, and catechol 1,2-dioxygenase CatA was upregulated by 3.47-fold in strain Z2 (Figures 2B,C). The RT-qPCR results showed that the gene encoding IaaA was upregulated 44- and 1.55-fold and that the gene encoding IacA was upregulated 1.51- and 28.9-fold in strains Z1 and Z2, respectively (Supplementary Figure S2). In addition, comparing the condition of mixed HNAD process with or without the addition of 10 mg/l IAA in proteomics, the key enzymes of IAA biosynthesis and degradation also significantly increased, revealing that the process of HNAD is closely related to IAA (Figure 2D).

Interestingly, although proteins related to IAA metabolism were upregulated to a certain extent in the mixed system, the level of difference was not the same in the two strains. In strain Z1, the increase in proteins associated with the *iaa* pathway was more significant, and the level differences were higher. In strain Z2, the expression of proteins in the *iac* pathway had a high degree of increase. These results indicated that the nitrogen removal systems of strains Z1 and Z2 were closely related to IAA metabolism, which may activate different metabolic pathways.

#### IAA Induced the Expression Levels of Key Enzymes in Denitrification

From another perspective, we highlighted the difference in the expression of proteins related to HNAD process in proteomics. Compared with the monoculture, the key enzymes of denitrification, nitrate reductase NarG and nitrite reductase NirS, were significantly upregulated, indicating a more efficient HNAD process in the coculture system (Figure 2B). In addition, we found that the expression levels of NarG and NirS in strains Z1 and Z2 were significantly upregulated under cocultured conditions with or without the addition of 10 mg/l exogenous IAA and were upregulated by 8.24- and 4.41-fold and 18.20- and 14.19-fold, respectively. Moreover, the upregulation levels of NarG and NirS in strain Z2 were higher than the upregulation levels of NarG and NirS in strain Z1 (Figures 2D,E).

In the RT-qPCR analysis, we used 10 mg/l exogenous IAA to explore its influence on the HNAD process, and the results revealed that NarG and NirS were significantly upregulated 3.34- and 3.73- and 13.67- and 4.47-fold by IAA induction, which was in agreement with the phenotypic experiment and proteomics results (Supplementary Figure S2).

### The *iaa* Pathway of Strain Z1 and the *iac* Pathway of Strain Z2 Played a Role in IAA Accumulation and Nitrogen Removal

#### Disturbance in the *iaa* or *iac* Pathway Inhibited Nitrogen Removal Rates of the Mixed Strains

To investigate the effect of the *iaa* and *iac* pathways on bacterial nitrogen removal, RNAi experiments were performed. The white and red fluorescent clones on the plate were selected to test the stability, the strains with the most stable silencing level of the target gene were selected as the interference type, and the nitrogen removal ability was explored in the following (Supplementary Figure S3).

**Figure 3:**
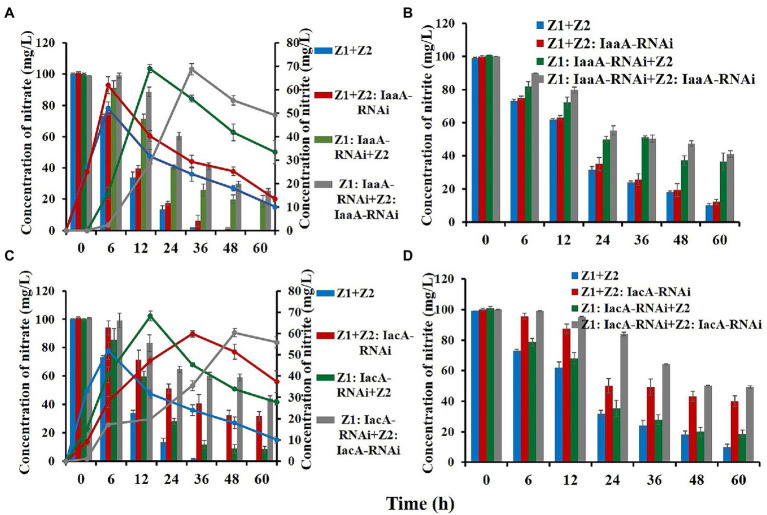
Nitrate and nitrite removal capability detection of the wild-type and RNAi strains. **(A,C)** Represent the removal of nitrate (column) and the change in nitrite content (curve) in the nitrate removal process. **(B,D)** Represent the change in nitrite concentration during its removal process. Values represent the mean ± standard deviation of three replicates.

Compared with the wild-type cocultured condition, all RNAi strains exhibited a degree of reduction in nitrogen removal ability, indicating that IAA metabolism was involved in and affected the nitrogen removal process as a key pathway (Figure 3; Table 1). For strain Z1, interference with the *iaa* pathway had a greater effect on nitrogen removal under mixed conditions, with nitrate and nitrite removal efficiencies dropping to 81.3% and 64.4% and removal rates decreasing to 1.65 and 1.07 mg/10^11^ h*colony forming units (cfu), respectively (Table 1). However, disturbance of the *iac* pathway of strain Z2 resulted in nitrate and nitrite removal efficiencies down to 69.3% and 59.9%, with removal rates of 1.93 and 0.99 mg/10^11^ h*cfu, respectively (Table 1). Meanwhile, in the cointerfered strains Z1 and Z2, HNAD levels in the system were further reduced (Table 1). The removal efficiencies of nitrate and nitrite decreased to 73.7% and 58.9% in the cointerfered strains of the *iaa* pathway, and the values dropped to 56.8% and 50.8% in the cointerfered strains of the *iac* pathway (Table 1).

**Table 1:** Nitrogen removal efficiency and IAA accumulation in wild-type and RNAi strains.

	Vmax (mg/10^11^ h*cfu)^**α**^	Efficiency (%)	IAA accumulation (mg/10^11^ cfu)
Nitrate removal system^β^			
Z1 + Z2	2.72 ± 0.35	100	58.1 ± 3.51
Z1: IaaA-RNAi + Z2	**1.65** ± 0.19^*^	81.3 ± 1.15	42.4 ± 0.85
Z1 + Z2: IaaA-RNAi	2.32 ± 0.41	100	51.7 ± 4.42
Z1: IaaA-RNAi + Z2: IaaA-RNAi	**1.07** ± 0.08^**^	**73.7** ± 0.57^*^	**39.7** ± 2.33^*^
Z1: IacA-RNAi + Z2	2.54 ± 0.14	91.5 ± 3.47	**30.6** ± 1.79^*^
Z1 + Z2: IacA-RNAi	**1.93** ± 0.28^*^	**69.3** ± 3.46^**^	**15.5** ± 1.16^**^
Z1: IacA-RNAi + Z2: IacA-RNAi	**1.57** ± 0.1^**^	**56.8** ± 2.42^**^	**7.8** ± 1.36^**^
Nitrite removal system			
Z1 + Z2	1.48 ± 0.13	100	54.3 ± 2.89
Z1: IaaA-RNAi + Z2	**1.07** ± 0.07^*^	**64.4** ± 1.91^*^	**36.9** ± 1.47^*^
Z1 + Z2: IaaA-RNAi	1.46 ± 0.11	87.8 ± 1.1	49.4 ± 1.65
Z1: IaaA-RNAi + Z2: IaaA-RNAi	**0.98** ± 0.09^**^	**58.9** ± 2.71^*^	**33.8** ± 1.01^*^
Z1: IacA-RNAi + Z2	1.38 ± 0.05	82.5 ± 2.28	**36.6** ± 0.68^*^
Z1 + Z2: IacA-RNAi	**0.99** ± 0.17^**^	**59.9** ± 2.92^*^	**21.2** ± 0.88^**^
Z1: IacA-RNAi + Z2: IacA-RNAi	**0.85** ± 0.07^**^	**50.8** ± 1.84^**^	**10.2** ± 1.13^**^

α The statistical significance is represented by stars between control groups (Z1 + Z2) and other groups, respectively.

β The value represents the mean of three replicates and the bold values indicate significant differences.

* *p* < 0.05;

** *p* < 0.01.

#### The Accumulation of IAA Was Decreased in RNAi Strains

When the *iac* pathway in strain Z2 was disturbed, the accumulation of IAA decreased to 15.5 and 21.2 mg/10^11^ cfu under the nitrate and nitrite removal processes, respectively. In addition, the concentration of IAA dropped to 7.8 and 10.2 mg/10^11^ cfu in the cointerfered strains of the *iac* pathway, and the amounts decreased to 39.7 and 33.8 mg/10^11^ cfu in the cointerfered strains of the *iaa* pathway (Table 1). Notably, in the “Z1: IaaA-RNAi + Z2” group, the accumulation of IAA did not decrease significantly, due to the existence of other regulatory mechanisms of nitrogen removal in the *iaa* metabolic pathway. In addition, analysis of all interference treatment groups showed that IAA accumulation decreased more significantly following interference of the *iac* pathway of strain Z2, revealing that strain Z2 may act as supplier and compensator of IAA in the cocultured system to ensure the high level of IAA in the condition (Table 1).

#### The Interference Resulted in Lower Levels of Expression of Key Nitrogen Removal Enzymes and the IAA Pathway

We used RT-qPCR to analyze the changes in the expression levels of key nitrogen removal enzymes and IAA metabolism between the RNAi and wild-type strains. The expression levels of NarG and NirS in “Z1: IaaA-RNAi” decreased by 1.96/1.45 and 2.22/2.63 times, respectively, during nitrate and nitrite removal, but there was no significant difference in the “Z2: IaaA-RNAi” group (Table 2). After interference with IacA, the expression levels of NarG and NirS in strain Z2 decreased by 3.57/1.75 and 4.17/5.26 times, respectively (Table 2). More importantly, the decreased expression level of key enzymes in the RNAi strains was consistent with the decreasing degree of denitrification ability of the system, which was further confirmed by the phenotype results. In addition, we investigated the differences in the expression levels of IpdC, IacA, and IaaA in the RNAi strains. Compared with the wild-type strains, the expression levels of IpdC of the IaaA-RNAi type of strain Z1 and the IacA-RNAi type of strain Z2 were significantly decreased to 2.04/9.09 and 1.38/1.85 times under nitrate and nitrite removal, respectively (Table 2). Moreover, interference with one IAA degradation pathway in strain Z1 could induce upregulation of key enzyme expression levels in the other IAA degradation pathway to a certain extent, while the loss of one IAA degradation pathway in strain Z2 inhibited the metabolic level of the other IAA degradation pathway.

**Table 2:** Upregulated ratio in relative gene expression levels between wild type and RNAi strains.

		Z1: IaaA-RNAi VS Z1	Z1: IacA-RNAi VS Z1	Z2: IaaA-RNAi VS Z2	Z2: IacA-RNAi VS Z2
Hao	Nitrate	−1.04 ± 0.09	1.04 ± 0.13	1.24 ± 0.1	−1.15 ± 0.03
	Nitrite	1.01 ± 0.06	1.1 ± 0.03	1.16 ± 0.09	−1.2 ± 0.08^*^
	IAA	−1.01 ± 0.11	1.2 ± 0.07	1.65 ± 0.12	1.01 ± 0.02
NarG	Nitrate	**−1.96** ± 0.01^*^	−1.03 ± 0.02	1.32 ± 0.24	**−3.57** ± 0.01^**^
	Nitrite	−1.45 ± 0.04	1.14 ± 0.05	1.19 ± 0.19	**−1.75** ± 0.03^*^
	IAA	−1.3 ± 0.09	1.07 ± 0.11	−1.02 ± 0.16	−1.2 ± 0.20
NirS	Nitrate	**−2.22** ± 0.04^**^	−1.13 ± 0.16	−1.15 ± 0.09	**−4.17** ± 0.02^**^
	Nitrite	**−2.63** ± 0.07^**^	1.21 ± 0.33	1.04 ± 0.21	**−5.26** ± 0.01^**^
	IAA	−1.59 ± 0.01	−1.04 ± 0.18	1.12 ± 0.07	**−2.08** ± 0.04^**^
IpdC	Nitrate	**−2.04** ± 0.09^**^	−1.11 ± 0.07	1.56 ± 0.63	**−9.09** ± 0.01^**^
	Nitrite	−1.38 ± 0.11	1.46 ± 0.28	1.21 ± 0.32	**−1.85** ± 0.12^*^
	IAA	−1.37 ± 0.1	−1.0 ± 0.06	1.04 ± 0.18	−1.23 ± 0.11
IaaA	Nitrate	NA^β^	1.6 ± 0.44	NA	−1.22 ± 0.13
	Nitrite	NA	**2.11** ± 0.32^**^	NA	−1.32 ± 0.04
	IAA	NA	1.49 ± 0.41	NA	**−1.75** ± 0.08^*^
IacA	Nitrate	1.21 ± 0.31	NA	−1.43 ± 0.57	NA
	Nitrite	**2.07** ± 0.28^**^	NA	−1.24 ± 0.33	NA
	IAA	**1.69** ± 0.19^*^	NA	−1.16 ± 0.11	NA

α The statistical significance is represented by stars between control groups (wild type) and other groups, respectively.

β NA, No data.

γ The value represents the mean of three replicates and the bold values indicate significant differences.

* *p* < 0.05;

** *p* < 0.01.

Taken together, the IAA pathways in strains Z1 and Z2 are different, including expression levels, and their effect on IAA accumulation and nitrogen removal efficiency. These results confirmed the conjecture of proteomics and RT-qPCR that strains Z1 and Z2 activated the hydrolyzation-redox pathway and decarboxylation pathway, respectively, under mixed conditions, which jointly promoted the rate of HNAD process.

### Other Metabolic Processes Associated With Nitrogen Removal

The HNAD process requires active energy metabolism and produces a large amount of oxidative stress, which also affects the cellular viability and rate of metabolism of strains to some degree. After interference with the key IAA metabolic pathway, the cell energy metabolism level decreased sharply, and the degree of reduction was more significant after interference with the *iac* pathway in strain Z2. When the *iac* pathway of strain Z2 was disturbed simultaneously, the NADH content decreased to 0.03 mg/g protein, and the ATP content dropped to 0.005 mg/g protein during nitrate and nitrite removal. The results indicated that strain Z2 played an important role in maintaining the energy metabolism level of the mixed system. In addition, the concentration of H_2_O_2_ in the system of the mixed strains was maintained at a lower level of 1.51 and 2.1 nmol/mg protein compared with the concentration of H_2_O_2_ in the monoculture, indicating that the oxidation pressure in the mixed cell was low, and the cell growth was strong. When the key metabolic pathways of IAA were disturbed, the H_2_O_2_ contents of all groups significantly accumulated to more than 5.0 nmol/mg protein, revealing that the oxidative pressure of cells in the system was enhanced, and the cell metabolism level was affected. Moreover, the inhibition of IAA pathway of strain Z1 had the greatest effect on intracellular oxidative stress. Meanwhile, we cultured wild-type and disturbed strains in response to nitrate and nitrite exposure and monitored the resistance of the strain to H_2_O_2_. The survival rates of the wild-type strain were above 90%, while the survival rates of strain Z1 with interference from the *iaa* pathway decreased to 32.4% and 27.75%, respectively, and the survival rates of strain Z2 with interference from the *iac* pathway dropped to 56.1% and 49.1%, respectively. The resistance of RNAi strain to oxidative stress decreased significantly, and the level of treatment group “Z1: IaaA-RNAi” was the most significant, which was consistent with the results of intracellular oxidative stress level. The results indicated that strain Z1 activated the hydrolyzation-redox pathway and improved the resistance of cells to oxidative stress (Supplementary Figure S4).

## Discussion

**Figure 4:**
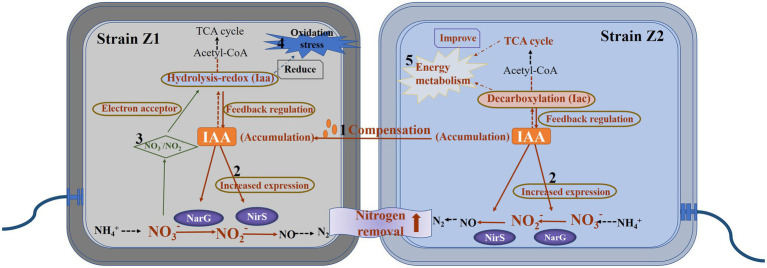
A hypothetical model of the IAA-mediated interaction between strains Z1 and Z2 for nitrogen removal. (1) The strain Z2 promoted the autologous Iac metabolic pathway, provided IAA for the strain Z1, and activated the Iaa metabolic pathway for strain Z1. (2) IAA induced the expression of key enzymes in denitrification in the nitrogen removal process. (3) The metabolic pathway of IAA in strain Z1 required nitrate and nitrite as electron acceptors. (4) The activation of Iaa pathway in strain Z1 reduced the level of intracellular oxidative stress. (5) Strain Z2 played a major role in promoting energy flow and maintaining metabolic levels.

In this study, the IAA metabolism of microorganisms was found to be closely related to the HNAD process using the mixed microbial agents. Through the analysis of a large number of experimental data in the early stage, the promotion of IAA metabolism on the HNAD process is revealed to be the result of multiple regulations, which can be summarized as the following perspectives (Figure 4). The IAA accumulation level of strain Z1 decreased significantly in the middle of nitrogen removal in single culture (Figure 1), which may be caused by the metabolic rate of IAA being higher than the synthesis rate. Under the cocultured conditions, the excess IAA produced by strain Z2 could be absorbed by strain Z1 to compensate for the deficiency of IAA in the cells, which directly induced the expression of denitrifying enzymes and further activated the overall metabolism level of IAA, thus greatly improving the HNAD ability of strain Z1. Next, the production and accumulation of IAA in strains Z1 and Z2 can induce the upregulated expression of NarG and NirS, key enzymes of denitrification, leading to the efficient implementation of the whole nitrogen removal process. In turn, nitrate and nitrite can induce the expression of key enzymes in the IAA synthesis and metabolism pathways, thus promoting the overall metabolism level of IAA in the system. Moreover, strains Z1 and Z2 enhanced two IAA metabolic pathways in the process of mixed nitrogen removal, that is, the hydrolyzation-redox pathway in strain Z1 was activated, and the level of decarboxylation degradation in strain Z2 was greatly increased. In addition, the *iaa* cluster, the key enzyme system in the hydrolyzation-redox pathway, will preferentially use nitrate and nitrite as electron receptors to form a complete electron transport chain and further activate the metabolism level of IAA, which is reflected mainly in strain Z1. Meanwhile, strain Z1 mainly regulated the level of oxidative stress and improved the resistance of cells to oxidative stress, and strain Z2 played a major role in maintaining a higher level of energy metabolism in the system. In general, strains Z1 and Z2 complemented and coordinated with each other in the mixed environment to promote the complete removal of nitrogen.

To further analyze the correlation between IAA metabolism and the microbial HNAD process, 10 strains belonging to *Enterobacter* and *Klebsiella* with or without *iaa* and *iac* pathways, respectively, were selected to explore the HNAD ability of bacteria under monocultured and cocultured conditions. The strains with a complete IAA metabolic pathway have higher nitrogen removal ability than the other strains in the monoculture condition, while the combination of *Enterobacter* with the *iaa* metabolic pathway and *Klebsiella* with the *iac* pathway significantly improved the nitrogen removal rate. All the above results further revealed the close relationship between IAA metabolism and nitrogen cycling in microbial cells.

Moreover, for wastewater treatment and agricultural soil remediation, mixed microbial agents play an effective and stable role in wastewater and can produce a large amount of IAA are used as PGPR, which proves the high efficiency and application potential and provides possibilities and prospects for improving root vitality, promoting the growth and development of the plant, and regulating the nitrogen cycle of root-soil.

## Conclusion

In this study, we explored the mechanism of superior HNAD ability of mixed strains Z1 and Z2 under cocultured conditions. Metabolomics, proteomics, and RT-qPCR analysis revealed that the process of HNAD was closely related to IAA metabolism. Interference analysis clarified that IAA activated two different metabolic pathways in strains Z1 and Z2 and directly induced the upregulation of key denitrifying enzymes. In addition, strain Z1 mainly regulated the level of oxidative stress, and strain Z2 played a major role in maintaining a higher level of energy metabolism.

## Data Availability Statement

The original contributions presented in the study are included in the article/Supplementary Material, further inquiries can be directed to the corresponding authors.

## Author Contributions

YZ: conceptualization, data curation, formal analysis, and roles/writing—original draft. QX: software, supervision, and validation. GW: funding acquisition and writing—review and editing. KS: conceptualization and writing—review and editing. All authors contributed to the article and approved the submitted version.

## Funding

This work was financially supported by the National Natural Science Foundation of China (31870086) and the Fundamental Research Funds for the Central Universities (project 2662022SKQD001).

## Conflict of Interest

The authors declare that the research was conducted in the absence of any commercial or financial relationships that could be construed as a potential conflict of interest.

## Publisher’s Note

All claims expressed in this article are solely those of the authors and do not necessarily represent those of their affiliated organizations, or those of the publisher, the editors and the reviewers. Any product that may be evaluated in this article, or claim that may be made by its manufacturer, is not guaranteed or endorsed by the publisher.

## Supplementary Material

The Supplementary Material for this article can be found online at: https://www.frontiersin.org/articles/10.3389/fmicb. 2022.929036/full#supplementary-material
