# Supplementary material for: Indole-Acetic Acid Promotes Ammonia Removal Through Heterotrophic Nitrification, Aerobic Denitrification With Mixed Enterobacter sp. Z1 and Klebsiella sp. Z2
Source: Front Microbiol. 2022 Jul 8;13:929036. doi: 10.3389/fmicb.2022.929036 (PMC9304994; doi:10.3389/fmicb.2022.929036)
Supplement: Supplementary file 1 [file Data_Sheet_1.DOCX]

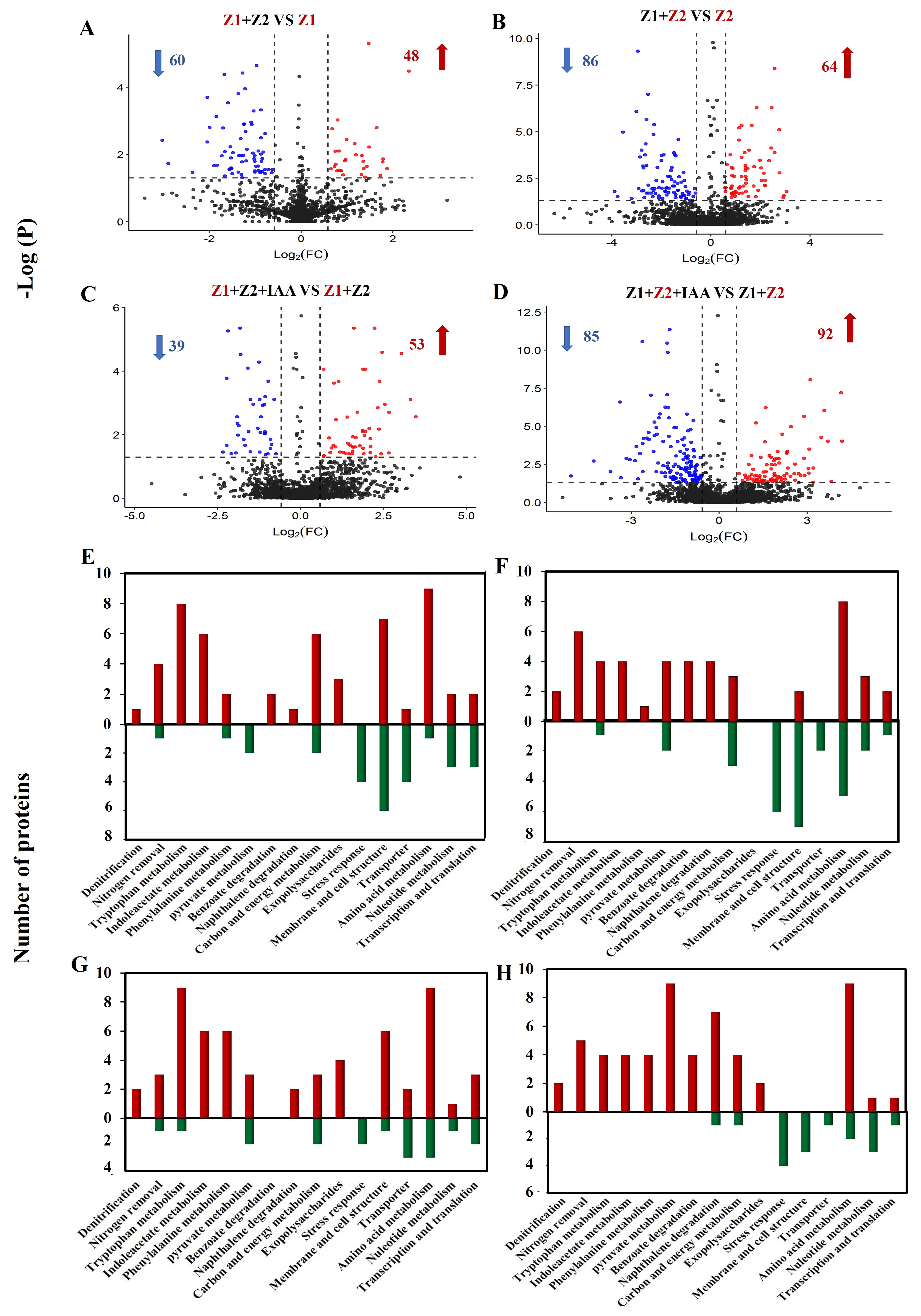


**Fig. S1** **Volcano map and the classification of proteins in COG categories based on iTRAQ proteomics analysis.** **A** and **E** represent differential proteins and metabolic pathways in strain Z1 between monocultures and cocultures under nitrate exposure, while **B** and **F** represent strain Z2. **C** and **G** represent the differential proteins and metabolic pathways of strain Z1 exposed to IAA, and **D** and **H** represent strain Z2. Red bars represent the upregulated proteins, and green bars represent the downregulated proteins.


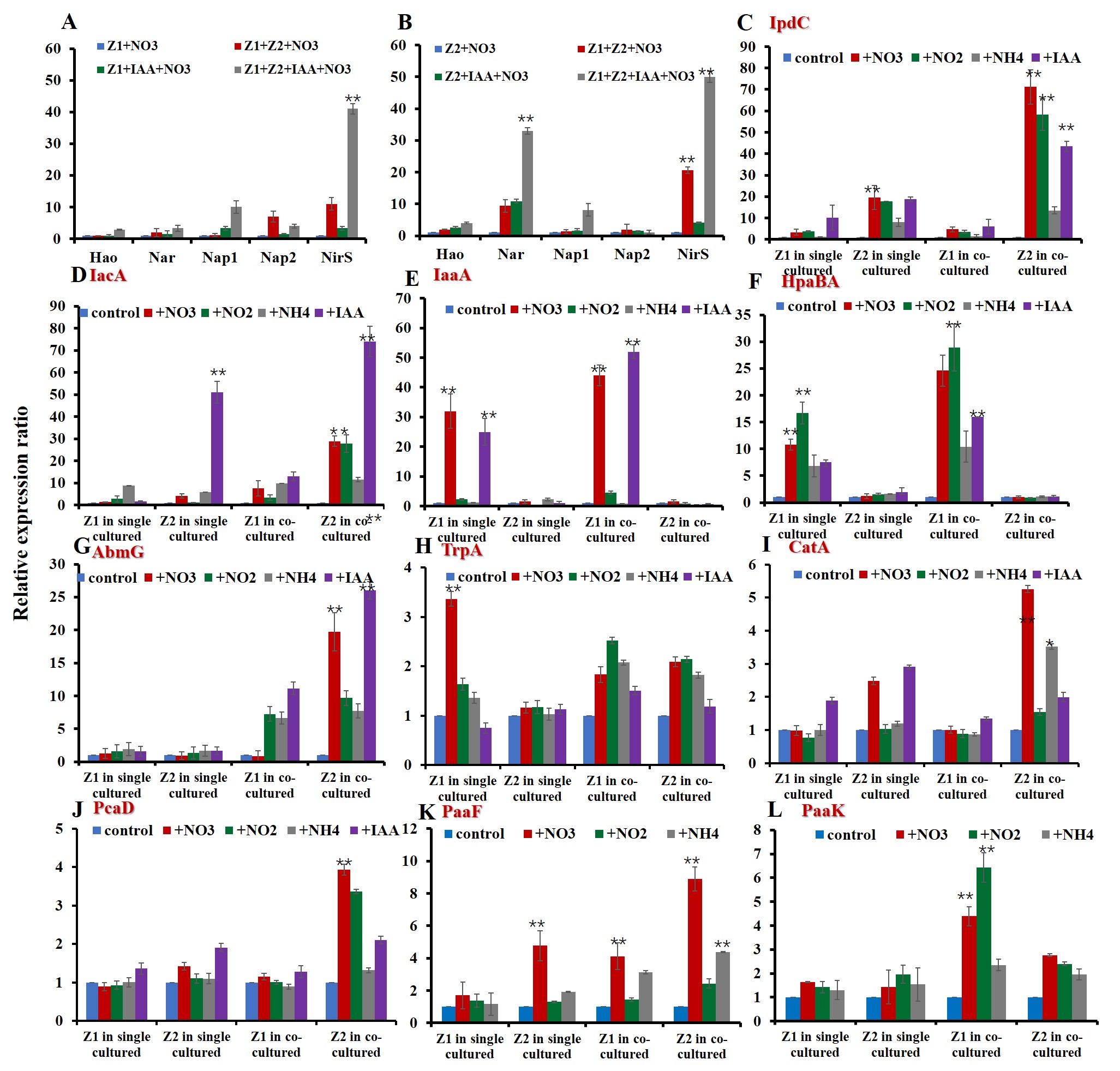


**Fig. S2 Expression levels of key proteins for nitrogen removal and IAA metabolism.** **A** and **B** represent the expression levels of key enzymes for nitrogen removal with the addition of nitrate and IAA. **C-L** represent the relative gene expression levels of key proteins for IAA metabolism of strains Z1 and Z2 under single or mixed culture conditions. The statistical significance is represented by stars (******p* < 0.05; *******p* < 0.01). Values represent the mean ± standard deviation of three replicates.


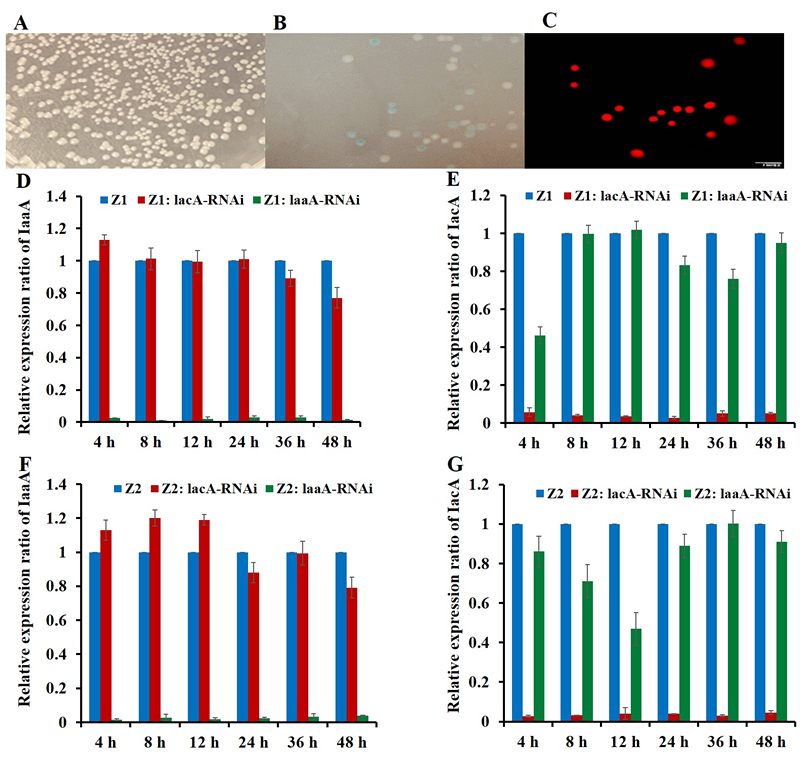


**Fig. S3** **Phenotypic validation and gene interference stability detection of RNAi strains**. **A** represents the wild type without plasmid insertion (white colonies without red fluorescence). **B** represents the strains with empty plasmid insertion (red fluorescence and blue colonies). **C** represents the RNAi strains with target fragment insertion (white colonies with red fluorescence). **D** and **F** represent the expression level of *iaaA* after RNA interference in strains Z1 and Z2, respectively. **E** and **G** represent the expression level of *iacA* after RNA interference in strains Z1 and Z2, respectively. Values represent the mean ± standard deviation of three replicates.


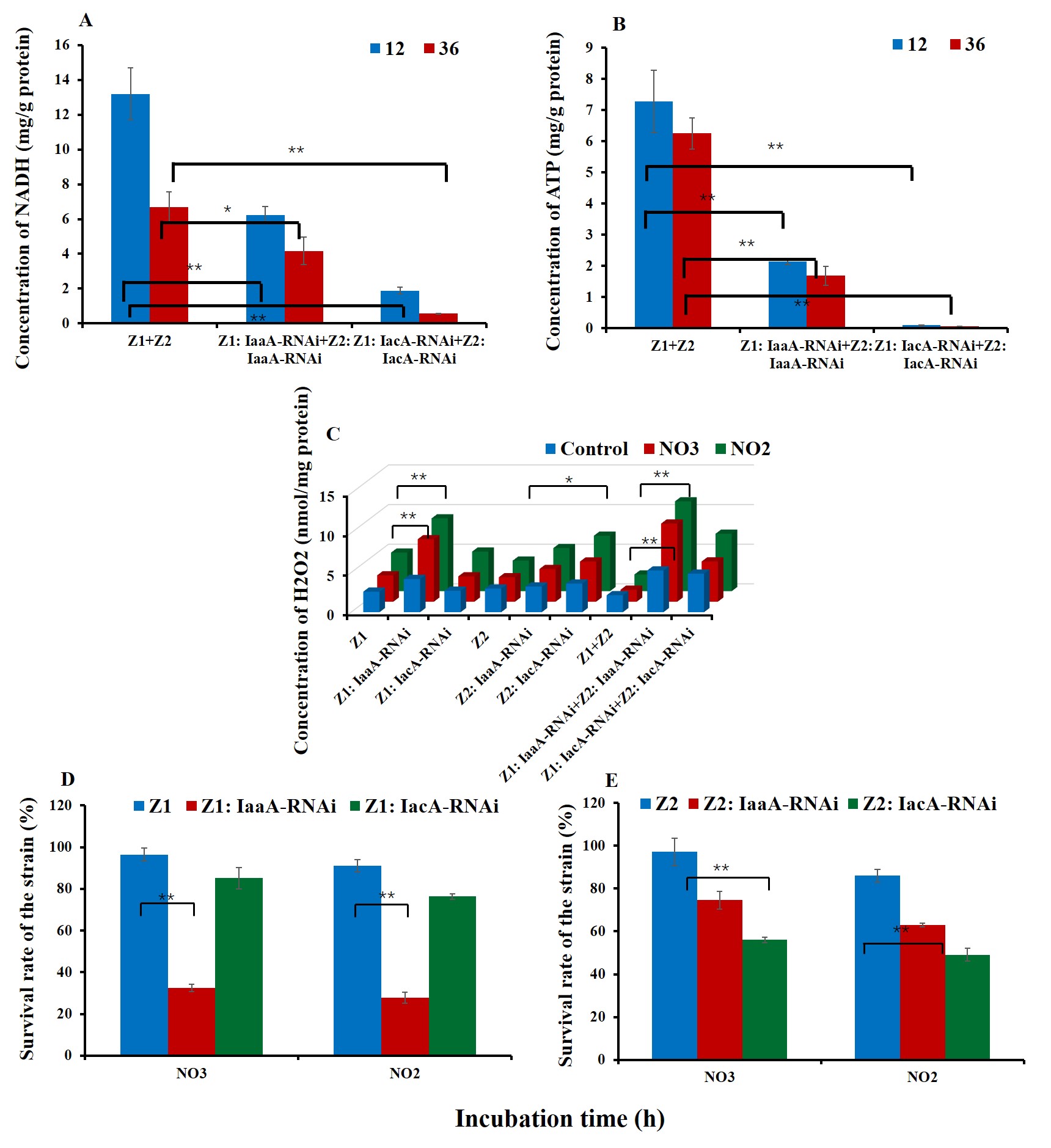


**Fig. S4 Energy substance and hydrogen peroxide content detection of wild-type and RNAi strains.** **A** and **B** represent the differences in intracellular NADH and ATP concentrations in the nitrate removal process at 12 and 36 h. **C** represents the accumulation level of hydrogen peroxide with the addition of nitrate and nitrite. **D** and **E** represent the tolerance to hydrogen peroxide under different nitrogen removal conditions between wild-type and RNAi strains. The statistical significance is represented by stars (******p* < 0.05; *******p* < 0.01). Values represent the mean ± standard deviation of three replicates.

Table S1. Characteristics, advantages, and disadvantages of different nitrogen removal technology.

| Technology | Characteristics | Advantages | Disadvantages | Reference |
| --- | --- | --- | --- | --- |
| **Anammox** | Low oxygen demand | Low sludge production; low greenhouse gas emissions | Low processing efficiency; treatment of low nitrogen wastewater | Chen et al., 2019; Wang et al., 2017 |
| **Simultaneous nitrification, anammox, and denitrification** | Partial oxidation of nitrification under oxygen restriction | No intermediate by-product remains | High sludge yield; Low processing efficiency; Poor stability | Wen et al., 2016 |
| **Simultaneous partial nitrification and denitrification** | Intermittent aeration | No intermediate by-product remains; Superior dominant flora | The reaction efficiency is very low when organic matter is insufficient | Huang et al., 2019 |
| **heterotrophic nitrification-aerobic denitrification** | Simultaneous nitrification and denitrification | High efficiency; lost cost; Low requirements for COD and oxygen concentrations | Few efficient bacteria have been found | Yang et al., 2019; Zhang et al., 2019 |

Table S2. List of nitrogen removal capabilities of HAND strains.

| Strains | Efficiency (%)/Rate (mg/L*h) | | | Reference |
| --- | --- | --- | --- | --- |
|  | Ammonia | Nitrate | Nitrite |  |
| *Acinetobacter* sp. JR1 | 98.5/4.22 | 91.1/NA | NA | Yang et al., 2019 |
| *Pseudomonas stutzeri* XL-2 | 96/NA | 97.9/NA | NA | Zhao et al., 2018 |
| *Pseudomonas tolaasii* Y-11 | 93.6/2.04 | 93.5/1.99 | 81.9/1.74 | He et al., 2016 |
| *Diaphorobacter* sp. SL-205 | 99.11/1.73 | 95.02/6.10 | 84.13/4.95 | Zhang et al., 2017 |
| *Enterobacter cloacae* CF-S27 | 98.0/11.6 | 99.0/5.1 | 58.9/1.2 | Padhi et al., 2017 |
| *Cupriavidus* sp. S1 | 99.7/10.4 | 98.0/8.6 | 99.8/8.4 | Sun et al., 2016 |
| *Enterobacter* sp. Z1+*Klebsiella* sp. Z2^α^ | 99.64/18.2 | 99.85/12.7 | 85/9.18 | Zhang et al., 2019 |

^α^The co-cultured strains were used in this study.

Table S3 Primers and RNA interference sequences designed in this study.

| **Primer** | **Sequence (5’-3’)** | **Description** |
| --- | --- | --- |
| Z1-16S F | CTGGAACTGAGACACGGTCC | For biomass analysis |
| Z1-16S R | AACCACAACACCTTCCTCCC |  |
| Z2-16S F | GCCTTCGGGTTGTAAAGCAC |  |
| Z2-16S R | CGCTTGCACCCTCCGTATTA |  |
| RT-*hao* F | CTACAGCTGGTGAGCGACG | For qRT-PCR |
| RT-*hao* R | TATAGGCCGCAGCACCTTTC |  |
| RT-*nar* F | TGTTTATCGGTTCGGTGGGG |  |
| RT-*nar* R | GCACCAGCAGCGTGATAATG |  |
| RT-*nap1* F | TGGGCTTCGAACCTGTTTCA |  |
| RT-*nap1* R | CCGCAATCATGGCTAACTGC |  |
| RT-*nap2* F | CCGGACTTCGATACCTTCCG |  |
| RT-*nap2* R | CGGCAATTTTTGCCAGCTCT |  |
| RT-*nirS* F | TATTCCCCGCCAGGAAAAGG |  |
| RT-*nirS* R | TGCCGTCGATAATGCTCTCC |  |
| RT-Z1-*ipdC* F | TGAAAGAGGTACCGATGGCG |  |
| RT-Z1-*ipdC* R | GGTATCGGTAAAACGCGTGC |  |
| RT-Z2-*ipdC* F | CCATGAAATCGACCGTGTGC |  |
| RT-Z2-*ipdC* R | CAAGCTGGTTTTCATCCGCC |  |
| RT-Z1-*iacA* F | CCAGGCGTTGAAATTGGTCG |  |
| RT-Z1-*iacA* R | GAGTTCGACGGCAGGGTAAT |  |
| RT-Z2-*iacA* F | CGCACGGAAAAAGAAGCCAT |  |
| RT-Z2-*iacA* R | TCGTGAGTGATGGCGAAGTC |  |
| RT-Z1-*iaaA* F | CATGGCATGACGCCAGTTTC |  |
| RT-Z1-*iaaA* R | GCTTTATCAAGAGCCACCGC |  |
| RT-Z2-*iaaA* F | CTGGTGCTGGAGAAGAGTCC |  |
| RT-Z2-*iaaA* R | CCATTTTCTGGCGTTCGTCG |  |
| RT-Z1-*hapBA* F | CGTATTCAGGAAACCGGGCT |  |
| RT-Z1-*hapBA* R | TGTTGTAGTGAGTCAGCGCC |  |
| RT-Z2-*hapBA* F | GCCTCCTATGAGATGGTCGC |  |
| RT-Z2-*hapBA* R | CAAAACCGCCTTCCATGGTC |  |
| RT-Z1-*abmG* F | CACTCGGTGATTTTTGGCGG |  |
| RT-Z1-*abmG* R | TACGCTTGAGGACGATGACG |  |
| RT-Z2-*abmG* F | TTTCGCCAATGTGTTGCTCG |  |
| RT-Z2-*abmG* R | TGGAGTCGATAATGCGTCCG |  |
| RT-Z1-*trpA* F | AGCACTGAAATCTCGCCAGG |  |
| RT-Z1-*trpA* R | AGCGTTGCGTTCTGGATAGT |  |
| RT-Z2-*trpA* F | TCGATGCGTTCTATGCCCAG |  |
| RT-Z2-*trpA* R | CGCAGTAAATCGTCATCCGC |  |
| RT-Z1-*catA* F | CGCAGTCAATCGTCATCCGC |  |
| RT-Z1-*catA* R | TGGCGTGCCAGATATTAACA |  |
| RT-Z2-*catA* F | CGCAGTAAATCGTCATCCGC |  |
| RT-Z2-*catA* R | TGGCGTGCCAGATATCAACA |  |
| RT-Z1-*pcaD* F | GCGTTAACCGCCCATTTCC |  |
| RT-Z1-*pcaD* R | CATCGAGATCCCGCAGAACC |  |
| RT-Z2-*pcaD* F | GCGTTAGCCGCCCATTTCC |  |
| RT-Z2-*pcaD* R | CATCGAGATCCCGTAGAACC |  |
| RT-Z1-*paaF* F | CCTGCCGGAAATTACCCTCG |  |
| RT-Z1-*paaF* R | CCGCCGGATGGATATCACTG |  |
| RT-Z2-*paaF* F | CCTGAACGATATCCGTCCCC |  |
| RT-Z2-*paaF* R | GACCAAAACGGGCGTTATCC |  |
| RT-Z1-*paaK* F | CCGACCGTCGTCGGTTATAC |  |
| RT-Z1-*paaK* R | TAAACGCTCTGCGCCATAGT |  |
| RT-Z2-*paaK* F | GCCAATATTGTCGCCCGTTC |  |
| RT-Z2-*paaK* R  RNAi | CTGTTTTTCGGTCTGTCCGC |  |
| Tac promoter | AAGCTTAATCATCGGCTCGTATAATGT | For RNAi analysis |
| Z1: IacA-RNAi | Tac + TACTACTAAAACTCGAGCTC |  |
| Z2: IacA-RNAi | Tac + TACTACTAAAACTCGAGCTC |  |
| Z1: IaaA-RNAi | Tac + TACCCTTACCAATTAGAGCTC |  |
| Z2: IaaA-RNAi | Tac + TACCCGTTCCGCCAAGAGCTC |  |

Table S4. The concentration of DO and COD during the nitrogen removal process.

|  | DO content (mg/L) | | COD content (mg/L) | |  |
| --- | --- | --- | --- | --- | --- |
| Ammonia removal system | 0 h | 60 h | 0 h | 60 h | |
| Strain Z1 | 8.52±0.21 | 8.34±0.12 | 58.3±2.3 | 54.3±1.7 | |
| Strain Z2 | 8.49±0.26 | 8.61±0.05 | 56.8±1.6 | 53.6±1.3 | |
| Strain Z1+Z2 | 8.27±0.12 | 8.39±0.21 | 57.3±2.9 | 53.1±2.2 | |
| Strain Z1+Z2 (+IAA) | 8.66±0.16 | 8.44±0.17 | 57.8±3.1 | 52.1±1.7 | |
| Nitrate removal system | 8.22±0.21 | 8.51±0.1 | 56.4±1.8 | 51.7±2.4 | |
| Strain Z1 | 8.38±0.11 | 8.39±0.24 | 56.8±2.5 | 52.5±1.6 | |
| Strain Z2 | 8.19±0.23 | 8.82±0.43 | 57.3±1.7 | 54.1±1.7 | |
| Strain Z1+Z2 | 8.62±0.24 | 8.23±0.09 | 59.2±2.2 | 53.4±1.9 | |
| Strain Z1+Z2 (+IAA) | 8.26±0.21 | 8.19±0.05 | 57.4±2.4 | 51.9±2.8 | |
| Nitrite removal system | 8.34±0.03 | 8.65±0.12 | 58.1±1.5 | 52.8±1.5 | |
| Strain Z1 | 8.46±0.13 | 8.48±0.11 | 57.4±1.3 | 52.5±2.1 | |
| Strain Z2 | 8.39±0.05 | 8.59±0.12 | 58.2±2.6 | 53.2±1.6 | |
| Strain Z1+Z2 | 8.38±0.11 | 8.38±0.22 | 59.1±3.1 | 51.9±1.3 | |
| Strain Z1+Z2 (+IAA) | 8.59±0.07 | 8.42±0.19 | 56.5±2.1 | 52.4±1.2 | |

^α^The value represents the mean of two replicates.

Table S5. The different expression of metabolites in extracellular metabolomics between strain Z1 and co-cultured condition.

| Metabolite | Formula | VIP value | FC(Y/X) | P value |
| --- | --- | --- | --- | --- |
| 3b-Hydroxy-5-cholenoic acid | C24H38O3 | 2.610 | 2406.906 | 2.12E-06 |
| 3-Indoleacetic Acid | C10H9NO2 | 1.623 | 105.738 | 9.8E-08 |
| Catechol | C6H6O2 | 3.102 | 22.432 | 1.36E-07 |
| Trehalose | C12H22O11 | 1.723 | 14.201 | 6.24E-05 |
| Anthranilate | C7H7O2 | 4.992 | 12.274 | 9.96E-07 |
| 4-OH-Phenylacetic acid | C8H8O2 | 0.999 | 10 | 0.001 |
| 4-Hydroxyflurbiprofen | C15H13FO3 | 1.073 | 4.537 | 6.86E-07 |
| Succinate | C4H4O4 | 1.698 | 4.177 | 8.24E-05 |
| Glutaminylphenylalanine | C14H19N3O4 | 2.606 | 3.215 | 2.42E-08 |
| Phenyllacticresent acid | C9H10O3 | 1.982 | 2.559 | 4.42E-10 |
| Tertatolol | C16H25NO2S | 1.020 | 2.503 | 2.19E-06 |
| Salicylic acid | C7H6O3 | 2.821 | 2.275 | 2.54E-05 |
| Isoleucyl-Glutamine | C11H21N3O4 | 1.731 | 2.086 | 6.05E-08 |
| Gln Asp Leu Glu | C20H33N5O10 | 4.659 | 1.944 | 3.38E-08 |
| gamma-Glutamylphenylalanine | C14H18N2O5 | 1.277 | 1.890 | 0.002 |
| Porphobilinogen | C10H14N2O4 | 1.770 | 1.846 | 2.72E-05 |
| (2S,3'S)-alpha-Amino-2-carboxy-5-oxo-1-pyrrolidinebutanoic acid | C9H14N2O5 | 1.161 | 1.806 | 2.46E-06 |
| LysoPE(0:0/22:6(4Z,7Z,10Z,13Z,16Z,19Z)) | C27H44NO7P | 1.746 | 1.585 | 0.001 |
| Gln Val Asp Glu | C19H31N5O10 | 3.716 | 1.577 | 1.71E-05 |
| N-Acetyl-DL-methionine | C7H13NO3S | 1.841 | 1.556 | 3.51E-06 |
| Pubescenol | C28H42O6 | 1.150 | 1.453 | 0.003 |
| 3a,4b,7a-Trihydroxy-5b-cholanoic acid | C24H40O5 | 1.985 | 1.419 | 2.86E-05 |
| D-Tryptophan | C11H12N2O2 | 3.307 | 1.416 | 0.001 |
| Glutamyltryptophan | C16H19N3O5 | 1.825 | 1.304 | 0.003 |
| Glutamylisoleucine | C11H20N2O5 | 1.850 | 1.239 | 0.010 |
| Asp Leu Ser Glu | C18H30N4O10 | 1.040 | 1.088 | 0.002 |
| Phytolaccoside F | C48H76O19 | 2.489 | 1.035 | 0.003 |
| Physagulin C | C30H38O9 | 1.176 | -1.168 | 0.030 |
| Cyclopassifloside VII | C37H62O13 | 5.526 | -1.221 | 0.001 |
| Glycylprolylhydroxyproline | C12H19N3O5 | 1.439 | -1.313 | 0.001 |
| Aspartyl-Arginine | C10H19N5O5 | 1.023 | -1.337 | 0.001 |
| 2-Deoxyribonic acid | C5H10O5 | 2.959 | -1.406 | 2.76E-07 |
| Indole-3-pyruvate | C11H8NO3 | 1.102 | -1.516 | 3.46E-07 |
| Kinetensin 4-7 | C26H37N9O6 | 2.140 | -1.564 | 3.01E-06 |
| (S)-2-Hydroxyglutarate | C5H8O5 | 2.681 | -2.623 | 2.54E-09 |
| Neuromedin B (1-3) | C12H22N4O5 | 2.523 | -3.003 | 8.2E-13 |
| N-(1-Deoxy-1-fructosyl)leucine | C12H23NO7 | 1.220 | -12.254 | 4.94E-16 |
| Cucurbitacin B | C32H46O8 | 1.120 | -12.594 | 1.25E-11 |
| Americine | C31H39N5O4 | 1.023 | -17.182 | 6.43E-13 |
| L-Tryptophan | C11H12N2O2 | 3.243 | -44.014 | 0.001 |
| DIPROTIN A | C17H31N3O4 | 1.067 | -333.333 | 1.61E-10 |

Table S6. The different expression proteins of strain Z1 in intracellular proteomics between single cultured and co-cultured conditions.

| Accession | Name | State | Regulated ratio | P value |
| --- | --- | --- | --- | --- |
| TYD07775.1 | phenylacetate-CoA oxygenase/reductase subunit PaaK | up | 6.234 | 0.001 |
| TYD07770.1 | 3-oxoadipyl-CoA thiolase IaaA | up | 5.071 | 0.001 |
| TYD05478.1 | type 1 fimbrial protein subunit FimA | up | 5.011 | 0.011 |
| TYD03078.1 | nitrate reductase subunit alpha NarG |  | 5.011 | 2.79E-05 |
| TYD07773.1 | 2-(1,2-epoxy-1,2-dihydrophenyl)acetyl-CoA isomerase | up | 4.742 | 0.011 |
| TYD03205.1 | tryptophan synthase subunit beta TrpBA | up | 4.742 | 0.001 |
| TYD05878.1 | DUF1479 domain-containing protein | up | 4.285 | 0.006 |
| TYD07867.1 | Re/Si-specific NAD(P)(+) transhydrogenase subunit alpha | up | 3.767 | 0.012 |
| TYD02844.1 | acetyl-CoA carboxylase biotin carboxylase subunit | up | 3.762 | 0.001 |
| TYD06497.1 | indolepyruvate decarboxylase IpdC | up | 3.664 | 0.001 |
| TYD01030.1 | formate dehydrogenase-N subunit alpha | up | 3.630 | 0.044 |
| TYD03218.1 | aldo/keto reductase | up | 3.467 | 0.007 |
| TYD06507.1 | long-chain fatty acid transporter FadL | up | 3.341 | 0.001 |
| TYD06508.1 | DUF406 family protein | up | 3.221 | 0.041 |
| TYD07839.1 | carboxypeptidase M32 | up | 3.162 | 0.026 |
| TYD03073.1 | porin OmpD | up | 3.133 | 0.014 |
| TYD02825.1 | NAD-dependent succinate-semialdehyde dehydrogenase | up | 3.047 | 8.68E-05 |
| TYD08353.1 | dimethylsulfoxide reductase subunit A | up | 2.964 | 0.005 |
| TYD01969.1 | cellulose biosynthesis cyclic di-GMP-binding regulatory protein BcsB | up | 2.937 | 0.001 |
| TYD04476.1 | methionine ABC transporter substrate-binding lipoprotein MetQ | up | 2.910 | 0.044 |
| TYD04713.1 | 4-hydroxyphenylacetate 3-monooxygenase, oxygenase component HpaBA | up | 2.890 | 2.53E-05 |
| TYD00814.1 | SDR family oxidoreductase | up | 2.805 | 0.046 |
| TYD01347.1 | phosphopyruvate hydratase | up | 2.754 | 0.029 |
| TYD08401.1 | lipoprotein | up | 2.728 | 0.008 |
| TYD08418.1 | nitroreductase NfsA | up | 2.728 | 0.029 |
| TYD01038.1 | glucose-1-phosphatase | up | 2.606 | 0.037 |
| TYD04528.1 | polynucleotide adenylyltransferase PcnB | up | 2.443 | 0.023 |
| TYD04019.1 | aldo/keto reductase | up | 2.398 | 0.039 |
| TYD01874.1 | aspartate-semialdehyde dehydrogenase | up | 2.133 | 0.026 |
| TYD06997.1 | type VI secretion system tube protein Hcp | up | 2.051 | 0.007 |
| TYD06648.1 | galactose/glucose ABC transporter substrate-binding protein MglB | up | 1.940 | 0.001 |
| TYD06994.1 | type VI secretion system contractile sheath large subunit | up | 1.923 | 8.56E-05 |
| TYD07008.1 | protein kinase | up | 1.905 | 0.013 |
| TYD06739.1 | glucose-1-phosphate thymidylyltransferase RfbA | up | 1.836 | 0.013 |
| TYD04041.1 | oxidative stress defense protein | up | 1.517 | 0.021 |
| TYD06531.1 | amidophosphoribosyltransferase | up | 1.515 | 0.039 |
| TYD05400.1 | ATP-dependent protease ATP-binding subunit ClpX | up | 1.514 | 0.001 |
| TYD04483.1 | YaeP family protein | up | 1.514 | 0.042 |
| TYD07733.1 | ATP-dependent RNA helicase HrpA | up | 1.514 | 0.025 |
| TYC99801.1 | 30S ribosomal protein S3 | up | 1.513 | 0.032 |
| TYD08358.1 | leucine-responsive transcriptional regulator Lrp | up | 1.513 | 0.003 |
| TYD08327.1 | chromosome partition protein MukB | up | 1.513 | 0.036 |
| TYD06555.1 | sugar phosphatase | up | 1.513 | 0.041 |
| TYD02700.1 | transcriptional regulator ExuR | up | 1.513 | 0.015 |
| TYD05446.1 | adenylate kinase | up | 1.513 | 0.004 |
| TYD06409.1 | IMP dehydrogenase | up | 1.513 | 0.009 |
| TYD03670.1 | signal recognition particle protein | up | 1.513 | 0.026 |
| TYC99807.1 | 50S ribosomal protein L5 | up | 1.503 | 0.042 |
| TYD05367.1 | tRNA guanosine(34) transglycosylase Tgt | down | -3.280 | 0.036 |
| TYD04622.1 | 4-hydroxy-3-methylbut-2-enyl diphosphate reductase | down | -3.311 | 0.041 |
| TYD05718.1 | glutamine--tRNA ligase | down | -3.467 | 0.043 |
| TYD07993.1 | 50S ribosomal protein L20 | down | -3.499 | 0.005 |
| TYD03834.1 | GTP diphosphokinase | down | -3.664 | 0.032 |
| TYC99794.1 | 30S ribosomal protein S10 | down | -3.837 | 0.013 |
| TYD02868.1 | glutamate synthase large subunit | down | -3.944 | 0.016 |
| TYC99809.1 | 30S ribosomal protein S8 | down | -4.017 | 0.016 |
| TYD06365.1 | phosphoribosylformylglycinamidine synthase | down | -4.055 | 0.027 |
| TYD00845.1 | dihydroxy-acid dehydratase | down | -4.246 | 0.043 |
| TYD03311.1 | peptide chain release factor 1 | down | -4.246 | 0.032 |
| TYD02758.1 | translation initiation factor IF-2 | down | -4.285 | 0.036 |
| TYD07992.1 | phenylalanine--tRNA ligase subunit alpha | down | -4.365 | 0.001 |
| TYC99865.1 | DNA-binding protein HU-alpha | down | -4.405 | 0.035 |
| TYD06410.1 | glutamine-hydrolyzing GMP synthase | down | -4.446 | 0.010 |
| TYD08070.1 | NADP-dependent isocitrate dehydrogenase | down | -4.613 | 0.007 |
| TYC99847.1 | 50S ribosomal protein L10 | down | -4.742 | 0.001 |
| TYD04920.1 | adenylosuccinate synthase | down | -4.830 | 0.040 |
| TYD02650.1 | RNA polymerase sigma factor RpoD | down | -4.830 | 1.11E-05 |
| TYD03666.1 | 50S ribosomal protein L19 | down | -4.920 | 0.010 |
| TYD06912.1 | pyruvate kinase | down | -4.965 | 0.023 |
| TYD04867.1 | FtsH protease activity modulator HflK | down | -5.105 | 0.028 |
| TYC99796.1 | 50S ribosomal protein L4 | down | -5.152 | 0.001 |
| TYD06948.1 | PTS mannose transporter subunit IIAB | down | -5.199 | 0.049 |
| TYD02685.1 | autoinducer 2 ABC transporter substrate-binding protein LsrB | down | -5.345 | 0.003 |
| TYD03261.1 | murein tripeptide/oligopeptide ABC transporter ATP binding protein OppF | down | -5.445 | 0.031 |
| TYD06755.1 | histidinol-phosphate transaminase | down | -5.445 | 0.018 |
| TYD06564.1 | NADH-quinone oxidoreductase subunit NuoF | down | -5.546 | 0.002 |
| TYD02710.1 | LysR family transcriptional regulator | down | -5.597 | 0.040 |
| TYD05769.1 | 2,3-diphosphoglycerate-dependent phosphoglycerate mutase | down | -5.807 | 0.011 |
| TYD06599.1 | DNA topoisomerase (ATP-hydrolyzing) subunit A | down | -5.861 | 0.002 |
| TYD06399.1 | histidine--tRNA ligase | down | -5.915 | 0.032 |
| TYD01049.1 | glutamate--ammonia ligase | down | -6.137 | 0.014 |
| TYD07995.1 | translation initiation factor IF-3 | down | -6.137 | 0.028 |
| TYC99821.1 | 50S ribosomal protein L17 | down | -6.194 | 0.007 |
| TYD02814.1 | 30S ribosomal protein S9 | down | -6.367 | 0.013 |
| TYD07781.1 | primary-amine oxidase | down | -6.546 | 0.018 |
| TYD08180.1 | trifunctional transcriptional regulator/proline dehydrogenase/L-glutamate gamma-semialdehyde dehydrogenase | down | -6.606 | 0.001 |
| TYD01057.1 | DNA polymerase I | down | -6.668 | 0.002 |
| TYD06730.1 | phosphomannomutase CpsG | down | -6.668 | 0.036 |
| TYD02759.1 | transcription termination/antitermination protein NusA | down | -6.729 | 0.012 |
| TYD02198.1 | F0F1 ATP synthase subunit beta | down | -6.854 | 0.002 |
| TYD04644.1 | two-component system response regulator ArcA | down | -6.982 | 0.007 |
| TYD07943.1 | pyruvate kinase PykF | down | -7.112 | 0.001 |
| TYC99805.1 | 50S ribosomal protein L14 | down | -7.177 | 0.001 |
| TYD00848.1 | ketol-acid reductoisomerase | down | -7.311 | 0.029 |
| TYD04631.1 | molecular chaperone DnaK | down | -7.447 | 7.33E-06 |
| TYD01048.1 | ribosome-dependent GTPase TypA | down | -7.516 | 1.27E-06 |
| TYD03926.1 | lysine--tRNA ligase | down | -7.798 | 0.007 |
| TYD00854.1 | transcription termination factor Rho | down | -7.943 | 0.001 |
| TYD02334.1 | protein-export chaperone SecB | down | -8.317 | 0.012 |
| TYD07970.1 | phosphoenolpyruvate synthase | down | -8.394 | 0.001 |
| TYD08366.1 | ATP-dependent Clp protease ATP-binding subunit | down | -8.472 | 0.007 |
| TYD03976.1 | methionine adenosyltransferase | down | -8.472 | 0.001 |
| TYD07991.1 | phenylalanine--tRNA ligase subunit beta | down | -8.790 | 0.040 |
| TYD04669.1 | purine-nucleoside phosphorylase | down | -8.790 | 0.046 |
| TYC99820.1 | DNA-directed RNA polymerase subunit alpha | down | -8.871 | 0.000 |
| TYD06757.1 | ATP phosphoribosyltransferase | down | -9.036 | 0.033 |
| TYD06476.1 | phosphoenolpyruvate-protein phosphotransferase PtsI | down | -9.204 | 0.004 |

Table S7. The different expression proteins of strain Z2 in intracellular proteomics between single cultured and co-cultured conditions.

| Accession | Name | State | Regulated ratio | P value |
| --- | --- | --- | --- | --- |
| TYC82955.1 | ferritin-like domain-containing protein | up | 8.279 | 0.015 |
| TYC82597.1 | nitrate reductase subunit alpha NarG | up | 8.279 | 0.005 |
| TYC83556.1 | SDR family oxidoreductase | up | 7.691 | 0.027 |
| TYC75555.1 | nitrite reductase large subunit NirS | up | 7.691 | 0.001 |
| TYC80613.1 | type 3 fimbria major subunit MrkA | up | 7.550 | 0.033 |
| TYC81307.1 | indolepyruvate decarboxylase IpdC | up | 7.550 | 0.008 |
| TYC82121.1 | TonB-dependent siderophore receptor | up | 6.823 | 0.001 |
| TYC79329.1 | glucose/quinate/shikimate family membrane-bound PQQ-dependent dehydrogenase | up | 6.760 | 7.82E-06 |
| TYC81843.1 | ubiquinone-dependent pyruvate dehydrogenase | up | 5.942 | 0.001 |
| TYC72201.1 | manganese catalase family protein | up | 5.942 | 4.1E-09 |
| TYC82431.1 | alpha,alpha-trehalose-phosphate synthase | up | 5.470 | 5.2E-07 |
| TYC68592.1 | lipocalin family protein | up | 5.419 | 7.57E-05 |
| TYC82958.1 | SDR family oxidoreductase | up | 5.081 | 0.001 |
| TYC83447.1 | tryptophan synthase subunit alpha TrpBA | up | 4.740 | 0.001 |
| TYC81056.1 | histidinol-phosphate transaminase | up | 4.549 | 0.004 |
| TYC82961.1 | YdeI family stress tolerance OB fold protein | up | 4.549 | 0.007 |
| TYC81090.1 | capsule assembly Wzi family protein | up | 4.508 | 0.007 |
| TYC81718.1 | NAD(P)H:quinone oxidoreductase | up | 4.385 | 0.001 |
| TYC83294.1 | phenylacetate--CoA ligase | up | 4.280 | 0.002 |
| TYC82731.1 | type I-E CRISPR-associated protein Cas7/Cse4/CasC | up | 4.111 | 0.004 |
| TYC81358.1 | transaldolase | up | 4.073 | 0.001 |
| TYC79514.1 | type 1 glutamine amidotransferase | up | 3.926 | 0.001 |
| TYC81275.1 | acetyl-CoA carboxylase, carboxyltransferase subunit | up | 3.870 | 0.017 |
| TYC83587.1 | ATP-independent periplasmic protein-refolding chaperone | up | 3.749 | 0.013 |
| TYC82544.1 | phosphoenolpyruvate carboxykinase (ATP) | up | 3.580 | 5.2E-07 |
| TYC75375.1 | acyl-CoA dehydrogenase/indole-3-acetate monooxygenase IacA | up | 3.580 | 0.001 |
| TYC82924.1 | catechol 1,2-dioxygenase CatA | up | 3.467 | 0.001 |
| TYC78272.1 | AsmA family protein | up | 3.357 | 0.001 |
| TYC68267.1 | hypothetical protein E4M18_027075 | up | 3.296 | 0.008 |
| TYC75595.1 | glycogen phosphorylase | up | 3.118 | 4.54E-06 |
| TYC82853.1 | VOC family protein | up | 2.844 | 0.029 |
| TYC82656.1 | DUF1852 domain-containing protein | up | 2.792 | 0.001 |
| TYC75597.1 | glucose-1-phosphate adenylyltransferase | up | 2.691 | 0.003 |
| TYC79250.1 | LPS assembly protein LptD | up | 2.675 | 0.017 |
| TYC82607.1 | UTP--glucose-1-phosphate uridylyltransferase GalU | up | 2.666 | 0.016 |
| TYC83037.1 | E3 ubiquitin--protein ligase | up | 2.666 | 0.005 |
| TYC82406.1 | alpha-amylase | up | 2.642 | 0.001 |
| TYC79546.1 | glutathione S-transferase family protein | up | 2.642 | 0.002 |
| TYC79201.1 | murein transglycosylase | up | 2.618 | 0.001 |
| TYC78585.1 | glyoxylate/hydroxypyruvate reductase GhrB | up | 2.618 | 0.001 |
| TYC82522.1 | alpha,alpha-trehalase | up | 2.570 | 0.001 |
| TYC82430.1 | trehalose-phosphatase | up | 2.477 | 0.008 |
| TYC79148.1 | "3,4-dihydroxyphenylacetate 2,3-dioxygenase " | up | 2.409 | 0.019 |
| TYC83304.1 | phenylacetic acid degradation bifunctional protein PaaZ | up | 2.365 | 0.002 |
| TYC78279.1 | cellulose biosynthesis cyclic di-GMP-binding regulatory protein BcsB | up | 2.365 | 4.54E-06 |
| TYC79187.1 | molecular chaperone OsmY | up | 2.365 | 0.001 |
| TYC83299.1 | phenylacetate-CoA oxygenase/reductase subunit | up | 2.259 | 0.009 |
| TYC81359.1 | transketolase | up | 2.197 | 6.25E-06 |
| TYC82674.1 | Fe-S cluster assembly protein SufD | up | 2.197 | 0.010 |
| TYC82657.1 | methionine synthase | up | 2.177 | 2.78E-05 |
| TYC75596.1 | glycogen synthase GlgA | up | 2.177 | 0.020 |
| TYC83494.1 | NAD(P)H-quinone oxidoreductase | up | 1.986 | 0.016 |
| TYC80871.1 | DUF883 domain-containing protein | up | 1.967 | 0.029 |
| TYC80429.1 | hypothetical protein E4M18_015345 | up | 1.949 | 0.020 |
| TYC81077.1 | mannose-1-phosphate guanylyltransferase/mannose-6-phosphate isomerase | up | 1.949 | 0.017 |
| TYC72577.1 | uroporphyrinogen-III C-methyltransferase | up | 1.914 | 0.002 |
| TYC80548.1 | Oxidative stress defense protein | up | 1.901 | 0.003 |
| TYC83303.1 | "1,2-phenylacetyl-CoA epoxidase subunit A " paaC | up | 1.896 | 0.018 |
| TYC79149.1 | 5-carboxymethyl-2-hydroxymuconate semialdehyde dehydrogenase | up | 1.828 | 0.001 |
| TYC83393.1 | peptide ABC transporter substrate-binding protein | up | 1.811 | 0.002 |
| TYC83295.1 | 3-oxoadipyl-CoA thiolase IaaA | up | 1.581 | 0.010 |
| TYC81076.1 | phosphomannomutase CpsG | up | 1.563 | 0.001 |
| TYC82677.1 | "L,D-transpeptidase family protein " | up | 1.520 | 0.010 |
| TYC82627.1 | 6-phosphofructokinase II | up | 1.506 | 0.016 |
| TYC79113.1 | tryptophan--tRNA ligase | down | -1.534 | 0.019 |
| TYC81213.1 | ribonucleoside-diphosphate reductase subunit alpha | down | -1.577 | 0.011 |
| TYC78879.1 | AAA family ATPase | down | -1.636 | 0.029 |
| TYC80286.1 | endopeptidase La | down | -1.667 | 0.048 |
| TYC81452.1 | flavodoxin-dependent (E)-4-hydroxy-3-methylbut-2-enyl-diphosphate synthase | down | -1.794 | 0.046 |
| TYC81211.1 | DNA topoisomerase (ATP-hydrolyzing) subunit A | down | -1.828 | 0.017 |
| TYC79018.1 | adenylosuccinate synthase | down | -1.862 | 0.023 |
| TYC81334.1 | Dyp-type peroxidase | down | -1.967 | 0.015 |
| TYC80753.1 | CTP synthase (glutamine hydrolyzing) | down | -1.986 | 0.038 |
| TYC81448.1 | ribosome biogenesis GTPase Der | down | -2.023 | 0.039 |
| TYC81282.1 | beta-ketoacyl-ACP synthase I | down | -2.041 | 0.008 |
| TYC72100.1 | outer membrane protein assembly factor BamA | down | -2.079 | 0.026 |
| TYC69241.1 | 50S ribosomal protein L5 | down | -2.098 | 0.004 |
| TYC81976.1 | molybdate ABC transporter substrate-binding protein | down | -2.177 | 0.026 |
| TYC81668.1 | ribonuclease E | down | -2.218 | 0.020 |
| TYC80940.1 | 50S ribosomal protein L19 | down | -2.280 | 0.005 |
| TYC81216.1 | glycerophosphodiester phosphodiesterase | down | -2.301 | 0.028 |
| TYC81778.1 | quinone-dependent dihydroorotate dehydrogenase | down | -2.301 | 0.033 |
| TYC78623.1 | "2,3-bisphosphoglycerate-independent phosphoglycerate mutase " | down | -2.432 | 0.002 |
| TYC79240.1 | carbamoyl-phosphate synthase large subunit | down | -2.477 | 2.62E-05 |
| TYC82630.1 | threonine--tRNA ligase | down | -2.500 | 0.013 |
| TYC72108.1 | 30S ribosomal protein S2 | down | -2.523 | 0.013 |
| TYC75448.1 | carbon starvation induced protein CsiD | down | -2.546 | 0.005 |
| TYC81954.1 | histidine ammonia-lyase | down | -2.691 | 0.001 |
| TYC81257.1 | acetate kinase | down | -2.691 | 0.023 |
| TYC72092.1 | acetyl-CoA carboxylase carboxyl transferase subunit | down | -2.691 | 0.035 |
| TYC80954.1 | ATP-dependent chaperone ClpB | down | -2.716 | 0.001 |
| TYC70210.1 | 50S ribosomal protein L1 | down | -2.766 | 0.004 |
| TYC79504.1 | polyribonucleotide nucleotidyltransferase | down | -2.818 | 0.008 |
| TYC79411.1 | acetyl-CoA carboxylase biotin carboxylase subunit | down | -2.818 | 0.001 |
| TYC81269.1 | lysine/arginine/ornithine ABC transporter substrate-binding protein ArgT | down | -2.818 | 0.018 |
| TYC78495.1 | isocitrate lyase | down | -2.897 | 0.008 |
| TYC81444.1 | IMP dehydrogenase | down | -2.978 | 0.001 |
| TYC81826.1 | leucine-responsive transcriptional regulator Lrp | down | -3.006 | 0.003 |
| TYC76079.1 | glutamate--ammonia ligase | down | -3.061 | 0.001 |
| TYC69247.1 | 30S ribosomal protein S3 | down | -3.147 | 0.002 |
| TYC79499.1 | transcription termination/antitermination protein NusA | down | -3.206 | 0.010 |
| TYC69243.1 | 50S ribosomal protein L14 | down | -3.265 | 0.018 |
| TYC80576.1 | peptide chain release factor 2 | down | -3.326 | 0.002 |
| TYC81357.1 | NADP-dependent oxaloacetate-decarboxylating malate dehydrogenase | down | -3.419 | 0.010 |
| TYC69248.1 | 50S ribosomal protein L22 | down | -3.451 | 0.024 |
| TYC79418.1 | rod shape-determining protein | down | -3.483 | 0.010 |
| TYC78179.1 | F0F1 ATP synthase subunit beta | down | -3.581 | 0.016 |
| TYC81443.1 | glutamine-hydrolyzing GMP synthase | down | -3.581 | 0.015 |
| TYC69229.1 | 30S ribosomal protein S4 | down | -3.749 | 0.001 |
| TYC76078.1 | ribosome-dependent GTPase TypA | down | -3.854 | 0.004 |
| TYC69252.1 | 50S ribosomal protein L4 | down | -3.854 | 0.038 |
| TYC81258.1 | phosphate acetyltransferase | down | -3.962 | 0.001 |
| TYC81302.1 | alanine transaminase | down | -3.962 | 0.010 |
| TYC79599.1 | RNA polymerase sigma factor RpoD | down | -3.999 | 0.001 |
| TYC72112.1 | DUF3461 family protein | down | -4.265 | 0.010 |
| TYC69254.1 | 30S ribosomal protein S10 | down | -4.265 | 0.037 |
| TYC69227.1 | 50S ribosomal protein L17 | down | -4.265 | 0.027 |
| TYC83493.1 | NADP-specific glutamate dehydrogenase | down | -4.466 | 0.001 |
| TYC81469.1 | IscS subfamily cysteine desulfurase | down | -4.592 | 0.004 |
| TYC69242.1 | 50S ribosomal protein L24 | down | -4.720 | 0.020 |
| TYC79690.1 | methionine adenosyltransferase | down | -4.808 | 0.011 |
| TYC81955.1 | urocanate hydratase | down | -4.897 | 4.17E-06 |
| TYC69253.1 | 50S ribosomal protein L3 | down | -4.988 | 1.39E-05 |
| TYC79585.1 | "1,3-propanediol dehydrogenase " | down | -4.988 | 0.014 |
| TYC81808.1 | 30S ribosomal protein S1 | down | -5.081 | 0.030 |
| TYC82005.1 | citrate (Si)-synthase | down | -5.176 | 0.011 |
| TYC82929.1 | formate dehydrogenase-N subunit alpha | down | -5.272 | 0.019 |
| TYC82581.1 | 3-deoxy-8-phosphooctulonate synthase | down | -5.728 | 0.019 |
| TYC79448.1 | 30S ribosomal protein S9 | down | -5.728 | 0.007 |
| TYC80405.1 | lysine decarboxylase CadA | down | -6.053 | 2.16E-06 |
| TYC79296.1 | cell division protein FtsZ | down | -6.053 | 0.019 |
| TYC78951.1 | "alpha,alpha-phosphotrehalase " | down | -6.053 | 0.001 |
| TYC69228.1 | DNA-directed RNA polymerase subunit alpha | down | -6.223 | 4.58E-05 |
| TYC70209.1 | 50S ribosomal protein L10 | down | -6.280 | 0.001 |
| TYC83257.1 | fumarate hydratase | down | -6.456 | 0.034 |
| TYC82454.1 | pyruvate kinase | down | -6.576 | 0.001 |
| TYC79233.1 | 4-hydroxy-3-methylbut-2-enyl diphosphate reductase | down | -6.576 | 0.013 |
| TYC82493.1 | PTS mannose transporter subunit IIAB | down | -6.760 | 0.001 |
| TYC78297.1 | ABC transporter substrate-binding protein | down | -6.886 | 0.015 |
| TYC72107.1 | elongation factor Ts | down | -6.950 | 9.88E-05 |
| TYC79574.1 | putrescine aminotransferase | down | -7.277 | 0.001 |
| TYC81220.1 | anaerobic glycerol-3-phosphate dehydrogenase subunit C | down | -7.551 | 0.011 |
| TYC79321.1 | bifunctional aconitate hydratase 2/2-methylisocitrate dehydratase | down | -7.762 | 4.8E-10 |
| TYC79224.1 | molecular chaperone DnaK | down | -8.053 | 8.21E-07 |
| TYC80339.1 | adenylate kinase | down | -9.162 | 0.037 |
| TYC69875.1 | NADP-dependent isocitrate dehydrogenase | down | -11.641 | 1.05E-05 |
| TYC81161.1 | galactose/glucose ABC transporter substrate-binding protein MglB | down | -13.614 | 0.030 |
| TYC70196.1 | phosphomethylpyrimidine synthase ThiC | down | -14.791 | 0.016 |

Table S8. The different expression proteins of strain Z1 in intracellular proteomics under co-cultured conditions without or with IAA.

| Accession | Name | State | Regulated ratio | P value |
| --- | --- | --- | --- | --- |
| TYD04713.1 | 4-hydroxyphenylacetate 3-monooxygenase, oxygenase component HpaBA | up | 17.864 | 2.53E-05 |
| TYD06497.1 | indolepyruvate decarboxylase IpdC | up | 11.066 | 0.002 |
| TYD02679.1 | putrescine aminotransferase | up | 9.908 | 0.001 |
| TYD07775.1 | phenylacetate-CoA oxygenase/reductase subunit PaaK | up | 8.810 | 0.001 |
| TYD03078.1 | nitrate reductase subunit alpha NarG | up | 8.241 | 2.79E-05 |
| TYD04041.1 | oxidative stress defense protein | up | 7.367 | 0.021 |
| TYD07770.1 | 3-oxoadipyl-CoA thiolase IaaA | up | 6.867 | 0.001 |
| TYC99812.1 | 30S ribosomal protein S5 | up | 6.309 | 0.037 |
| TYD05777.1 | molybdate ABC transporter substrate-binding protein | up | 5.807 | 0.001 |
| TYD07783.1 | aldehyde dehydrogenase family protein | up | 5.395 | 0.039 |
| TYD02684.1 | 3-hydroxy-5-phosphonooxypentane-2,4-dione thiolase | up | 5.199 | 0.001 |
| TYC99817.1 | 30S ribosomal protein S13 | up | 5.152 | 0.006 |
| TYD06393.1 | alpha-2-macroglobulin family protein | up | 5.011 | 0.001 |
| TYD01049.1 | glutamate--ammonia ligase | up | 4.786 | 0.049 |
| TYD08180.1 | trifunctional transcriptional regulator/proline dehydrogenase/L-glutamate gamma-semialdehyde dehydrogenase | up | 4.698 | 4.46E-06 |
| TYD01828.1 | nitrite reductase large subunit NirS | up | 4.411 | 0.013 |
| TYD02352.1 | aldehyde dehydrogenase | up | 4.325 | 0.022 |
| TYD02339.1 | alpha-hydroxy-acid oxidizing protein | up | 4.285 | 0.037 |
| TYD01935.1 | bifunctional UDP-4-amino-4-deoxy-L-arabinose formyltransferase/UDP-glucuronic acid oxidase ArnA | up | 4.207 | 0.006 |
| TYD03202.1 | anthranilate synthase TrpC | up | 3.872 | 8.73E-05 |
| TYD07700.1 | ferritin-like domain-containing protein | up | 3.872 | 0.007 |
| TYD01434.1 | DUF305 domain-containing protein | up | 3.801 | 0.010 |
| TYC99810.1 | 50S ribosomal protein L6 | up | 3.732 | 0.040 |
| TYD02825.1 | NAD-dependent succinate-semialdehyde dehydrogenase | up | 3.698 | 8.73E-05 |
| TYD04706.1 | 5-carboxymethyl-2-hydroxymuconate semialdehyde dehydrogenase | up | 3.698 | 0.026 |
| TYD02844.1 | acetyl-CoA carboxylase biotin carboxylase subunit | up | 3.664 | 0.008 |
| TYD02704.1 | DUF883 domain-containing protein | up | 3.664 | 0.007 |
| TYD01874.1 | aspartate-semialdehyde dehydrogenase | up | 3.351 | 0.022 |
| TYD07781.1 | primary-amine oxidase | up | 3.280 | 0.001 |
| TYD05678.1 | alpha/beta hydrolase | up | 3.162 | 0.012 |
| TYD05403.1 | peptidylprolyl isomerase | up | 3.133 | 0.024 |
| TYD04496.1 | outer membrane protein assembly factor BamA | up | 3.047 | 4.46E-06 |
| TYD06390.1 | 3-mercaptopyruvate sulfurtransferase | up | 3.019 | 0.024 |
| TYD01648.1 | CsbD family protein | up | 2.964 | 0.033 |
| TYD00817.1 | MBL fold metallo-hydrolase | up | 2.964 | 0.011 |
| TYD04888.1 | lipocalin family protein | up | 2.964 | 0.024 |
| TYD06508.1 | DUF406 family protein | up | 2.937 | 0.040 |
| TYD03940.1 | glycine cleavage system aminomethyltransferase GcvT | up | 2.805 | 0.021 |
| TYD04504.1 | 30S ribosomal protein S2 | up | 2.754 | 0.040 |
| TYD05718.1 | glutamine--tRNA ligase | up | 2.679 | 0.018 |
| TYD04602.1 | LPS assembly protein LptD | up | 2.654 | 0.002 |
| TYD06460.1 | transketolase | up | 2.558 | 0.038 |
| TYD06520.1 | beta-ketoacyl-ACP synthase I | up | 2.443 | 0.036 |
| TYD04612.1 | carbamoyl-phosphate synthase large subunit | up | 2.269 | 0.034 |
| TYD06648.1 | galactose/glucose ABC transporter substrate-binding protein MglB | up | 2.208 | 0.001 |
| TYD03760.1 | alanine--tRNA ligase | up | 2.089 | 0.003 |
| TYD08193.1 | bifunctional glucose-1-phosphatase/inositol phosphatase | up | 2.070 | 0.024 |
| TYD00812.1 | alcohol dehydrogenase catalytic domain-containing protein | up | 2.013 | 0.001 |
| TYC99814.1 | 50S ribosomal protein L15 | up | 1.905 | 0.026 |
| TYD06997.1 | type VI secretion system tube protein Hcp | up | 1.853 | 0.036 |
| TYD07007.1 | type VI secretion system ATPase TssH | up | 1.819 | 0.012 |
| TYD06594.1 | glycerophosphodiester phosphodiesterase | up | 1.629 | 0.045 |
| TYD07970.1 | phosphoenolpyruvate synthase | up | 1.614 | 8.73E-05 |
| TYD00854.1 | transcription termination factor Rho | down | -1.674 | 0.040 |
| TYD04631.1 | molecular chaperone DnaK | down | -1.737 | 0.001 |
| TYD05445.1 | molecular chaperone HtpG | down | -1.836 | 0.020 |
| TYD05700.1 | leucine--tRNA ligase | down | -1.870 | 0.026 |
| TYD03261.1 | murein tripeptide/oligopeptide ABC transporter ATP binding protein OppF | down | -1.870 | 0.013 |
| TYD08050.1 | PrkA family serine protein kinase | down | -1.958 | 0.001 |
| TYD03927.1 | peptide chain release factor 2 | down | -1.976 | 0.040 |
| TYD02698.1 | glucuronate isomerase | down | -2.070 | 0.042 |
| TYD04669.1 | purine-nucleoside phosphorylase | down | -2.089 | 0.001 |
| TYD03976.1 | methionine adenosyltransferase | down | -2.089 | 0.009 |
| TYD04563.1 | preprotein translocase subunit SecA | down | -2.108 | 0.008 |
| TYD06476.1 | phosphoenolpyruvate-protein phosphotransferase PtsI | down | -2.128 | 0.009 |
| TYD06676.1 | methionine--tRNA ligase | down | -2.128 | 0.001 |
| TYD08070.1 | NADP-dependent isocitrate dehydrogenase | down | -2.208 | 0.001 |
| TYD05769.1 | 2,3-diphosphoglycerate-dependent phosphoglycerate mutase | down | -2.290 | 0.002 |
| TYD04672.1 | deoxyribose-phosphate aldolase | down | -2.312 | 0.008 |
| TYD04547.1 | pyruvate dehydrogenase complex dihydrolipoyllysine-residue acetyltransferase | down | -2.355 | 0.001 |
| TYD07675.1 | cobalamin-independent methionine synthase II family protein | down | -2.355 | 0.034 |
| TYD03651.1 | ATP-dependent chaperone ClpB | down | -2.376 | 5.17E-05 |
| TYD07996.1 | threonine--tRNA ligase | down | -2.443 | 0.006 |
| TYD06564.1 | NADH-quinone oxidoreductase subunit NuoF | down | -2.679 | 0.001 |
| TYD06371.1 | serine hydroxymethyltransferase | down | -2.857 | 0.001 |
| TYD04670.1 | phosphopentomutase | down | -2.857 | 0.034 |
| TYD05741.1 | citrate synthase | down | -2.964 | 0.007 |
| TYD02765.1 | ATP-dependent zinc metalloprotease FtsH | down | -3.019 | 8.06E-05 |
| TYD06478.1 | cysteine synthase A | down | -3.191 | 0.021 |
| TYD03124.1 | pyruvate:ferredoxin (flavodoxin) oxidoreductase | down | -3.280 | 0.014 |
| TYD03823.1 | assimilatory sulfite reductase (NADPH) hemoprotein subunit | down | -3.499 | 3E-05 |
| TYD05673.1 | alkyl hydroperoxide reductase subunit C | down | -3.531 | 4.46E-06 |
| TYD06562.1 | NADH-quinone oxidoreductase subunit C/D | down | -3.630 | 0.005 |
| TYD04576.1 | peptidoglycan glycosyltransferase FtsI | down | -3.698 | 0.010 |
| TYD06469.1 | sulfate ABC transporter substrate-binding protein | down | -3.732 | 0.004 |
| TYD00890.1 | 5-methyltetrahydropteroyltriglutamate--homocysteine S-methyltransferase | down | -3.767 | 0.002 |
| TYD01565.1 | acetate--CoA ligase | down | -3.872 | 0.037 |
| TYD05373.1 | nucleoside-specific channel-forming protein Tsx | down | -4.246 | 0.040 |
| TYD05746.1 | The 2-oxoglutarate dehydrogenase E1 component | down | -4.570 | 5.41E-06 |
| TYD03822.1 | phosphoadenosine phosphosulfate reductase | down | -4.698 | 0.001 |
| TYD00892.1 | uridine phosphorylase | down | -4.698 | 0.021 |
| TYD03818.1 | sulfate adenylyltransferase subunit CysN | down | -5.058 | 0.035 |

Table S9. The different expression proteins of strain Z2 in intracellular proteomics under co-cultured conditions without or with IAA.

| Accession | Name | State | Regulated ratio | P value |
| --- | --- | --- | --- | --- |
| TYC79574.1 | putrescine aminotransferase | up | 18.197 | 9.56E-05 |
| TYC82597.1 | nitrate reductase subunit alpha NarG | up | 18.197 | 0.034 |
| TYC75555.1 | nitrite reductase large subunit NirS | up | 14.190 | 0.012 |
| TYC70190.1 | DNA-binding protein HU-alpha | up | 14.190 | 0.042 |
| TYC78495.1 | isocitrate lyase | up | 13.061 | 9.56E-05 |
| TYC72540.1 | fatty acid oxidation complex subunit alpha FadB | up | 12.022 | 9.29E-07 |
| TYC81307.1 | indolepyruvate decarboxylase IpdC | up | 12.022 | 0.005 |
| TYC70209.1 | 50S ribosomal protein L10 | up | 11.168 | 5.26E-05 |
| TYC82924.1 | catechol 1,2-dioxygenase CatA | up | 9.762 | 0.019 |
| TYC81905.1 | glutamine ABC transporter substrate-binding protein GlnH | up | 9.289 | 0.005 |
| TYC79601.1 | 30S ribosomal protein S21 | up | 9.204 | 0.048 |
| TYC81706.1 | trifunctional transcriptional regulator/proline dehydrogenase/L-glutamate gamma-semialdehyde dehydrogenase | up | 8.629 | 8.92E-09 |
| TYC81808.1 | 30S ribosomal protein S1 | up | 8.394 | 0.001 |
| TYC79149.1 | 5-carboxymethyl-2-hydroxymuconate semialdehyde dehydrogenase | up | 8.317 | 0.011 |
| TYC69254.1 | 30S ribosomal protein S10 | up | 8.241 | 0.005 |
| TYC75375.1 | acyl-CoA dehydrogenase/indole-3-acetate monooxygenase IacA | up | 7.920 | 0.034 |
| TYC83294.1 | phenylacetate--CoA ligase | up | 7.711 | 0.005 |
| TYC81292.1 | acetyl-CoA C-acyltransferase FadI | up | 7.516 | 0.016 |
| TYC83447.1 | tryptophan synthase subunit alpha TrpBA | up | 7.516 | 0.001 |
| TYC79240.1 | carbamoyl-phosphate synthase large subunit | up | 7.447 | 2.25E-06 |
| TYC69242.1 | 50S ribosomal protein L24 | up | 6.546 | 0.012 |
| TYC80548.1 | oxidative stress defense protein | up | 6.309 | 0.001 |
| TYC76079.1 | glutamate--ammonia ligase | up | 6.025 | 0.003 |
| TYC79447.1 | 50S ribosomal protein L13 | up | 6.025 | 0.034 |
| TYC79448.1 | 30S ribosomal protein S9 | up | 5.915 | 0.012 |
| TYC82052.1 | LPS assembly lipoprotein LptE | up | 5.780 | 0.042 |
| TYC78879.1 | AAA family ATPase | up | 5.445 | 1.07E-05 |
| TYC79593.1 | YfdX family protein | up | 5.395 | 0.019 |
| TYC69228.1 | DNA-directed RNA polymerase subunit alpha | up | 5.058 | 0.005 |
| TYC69244.1 | 30S ribosomal protein S17 | up | 5.058 | 0.047 |
| TYC83023.1 | aspartate-semialdehyde dehydrogenase | up | 5.055 | 0.029 |
| TYC69229.1 | 30S ribosomal protein S4 | up | 5.011 | 0.001 |
| TYC72092.1 | acetyl-CoA carboxylase carboxyl transferase subunit alpha | up | 4.920 | 0.001 |
| TYC79200.1 | energy-dependent translational throttle protein EttA | up | 4.920 | 0.001 |
| TYC79411.1 | acetyl-CoA carboxylase biotin carboxylase subunit | up | 4.920 | 0.001 |
| TYC82544.1 | phosphoenolpyruvate carboxykinase (ATP) | up | 4.920 | 0.012 |
| TYC79571.1 | autoinducer 2 ABC transporter substrate-binding protein LsrB | up | 4.742 | 0.029 |
| TYC83051.1 | ribulose-phosphate 3-epimerase | up | 4.655 | 0.017 |
| TYC69253.1 | 50S ribosomal protein L3 | up | 4.446 | 3.31E-05 |
| TYC78243.1 | undecaprenyl-phosphate 4-deoxy-4-formamido-L-arabinose transferase | up | 4.365 | 0.029 |
| TYC79581.1 | glycerol dehydrogenase | up | 4.325 | 0.042 |
| TYC75528.1 | 30S ribosomal protein S7 | up | 4.246 | 0.023 |
| TYC78242.1 | bifunctional UDP-4-amino-4-deoxy-L-arabinose formyltransferase/UDP-glucuronic acid oxidase ArnA | up | 4.130 | 0.002 |
| TYC80940.1 | 50S ribosomal protein L19 | up | 4.092 | 0.001 |
| TYC82634.1 | phenylalanine--tRNA ligase subunit alpha | up | 4.055 | 0.019 |
| TYC81976.1 | molybdate ABC transporter substrate-binding protein | up | 3.981 | 0.001 |
| TYC81826.1 | leucine-responsive transcriptional regulator Lrp | up | 3.944 | 0.001 |
| TYC79572.1 | 3-hydroxy-5-phosphonooxypentane-2,4-dione thiolase | up | 3.908 | 0.028 |
| TYC79304.1 | GMP reductase | up | 3.837 | 0.034 |
| TYC69252.1 | 50S ribosomal protein L4 | up | 3.801 | 0.040 |
| TYC79500.1 | translation initiation factor IF-2 | up | 3.732 | 0.017 |
| TYC76078.1 | ribosome-dependent GTPase TypA | up | 3.732 | 0.001 |
| TYC79599.1 | RNA polymerase sigma factor RpoD | up | 3.732 | 0.003 |
| TYC79233.1 | 4-hydroxy-3-methylbut-2-enyl diphosphate reductase | up | 3.732 | 0.036 |
| TYC81257.1 | acetate kinase | up | 3.698 | 0.017 |
| TYC83305.1 | primary-amine oxidase | up | 3.630 | 0.001 |
| TYC81499.1 | elongation factor 4 | up | 3.499 | 0.042 |
| TYC82631.1 | translation initiation factor IF-3 | up | 3.499 | 0.028 |
| TYC72108.1 | 30S ribosomal protein S2 | up | 3.467 | 0.003 |
| TYC82635.1 | phenylalanine--tRNA ligase subunit beta | up | 3.435 | 0.006 |
| TYC81448.1 | ribosome biogenesis GTPase Der | up | 3.404 | 0.001 |
| TYC81282.1 | beta-ketoacyl-ACP synthase I | up | 3.404 | 0.033 |
| TYC70210.1 | 50S ribosomal protein L1 | up | 3.372 | 0.032 |
| TYC81291.1 | fatty acid oxidation complex subunit alpha FadJ | up | 3.280 | 0.001 |
| TYC81878.1 | 30S ribosomal protein S12 methylthiotransferase RimO | up | 3.133 | 0.045 |
| TYC78623.1 | 2,3-bisphosphoglycerate-independent phosphoglycerate mutase | up | 3.076 | 0.003 |
| TYC80753.1 | CTP synthase (glutamine hydrolyzing) | up | 3.019 | 0.009 |
| TYC83299.1 | phenylacetate-CoA oxygenase/reductase subunit PaaK | up | 3.010 | 0.001 |
| TYC82650.1 | phosphoenolpyruvate synthase | up | 2.937 | 0.001 |
| TYC81443.1 | glutamine-hydrolyzing GMP synthase | up | 2.937 | 0.019 |
| TYC79499.1 | transcription termination/antitermination protein NusA | up | 2.910 | 0.035 |
| TYC75589.1 | maltodextrin phosphorylase | up | 2.857 | 0.010 |
| TYC80937.1 | signal recognition particle protein | up | 2.779 | 0.036 |
| TYC79418.1 | rod shape-determining protein | up | 2.779 | 0.034 |
| TYC76008.1 | argininosuccinate lyase | up | 2.754 | 0.042 |
| TYC81319.1 | NAD-dependent DNA ligase LigA | up | 2.679 | 0.001 |
| TYC69236.1 | 30S ribosomal protein S5 | up | 2.654 | 0.039 |
| TYC69241.1 | 50S ribosomal protein L5 | up | 2.606 | 0.018 |
| TYC69234.1 | 50S ribosomal protein L15 | up | 2.582 | 0.023 |
| TYC79142.1 | 4-hydroxyphenylacetate 3-monooxygenase, oxygenase component | up | 2.490 | 0.023 |
| TYC79579.1 | dihydroxyacetone kinase subunit DhaK | up | 2.443 | 0.031 |
| TYC71047.1 | tellurium resistance membrane protein TerB | up | 2.421 | 0.016 |
| TYC70207.1 | DNA-directed RNA polymerase subunit beta | up | 2.376 | 6.06E-06 |
| TYC78985.1 | autotransporter assembly complex protein TamA | up | 2.376 | 0.032 |
| TYC72100.1 | outer membrane protein assembly factor BamA | up | 2.312 | 0.018 |
| TYC69237.1 | 50S ribosomal protein L18 | up | 2.290 | 0.041 |
| TYC81893.1 | ABC-F family ATPase | up | 2.032 | 0.020 |
| TYC83295.1 | 3-oxoadipyl-CoA thiolase IaaA | up | 2.013 | 0.016 |
| TYC82679.1 | pyruvate kinase PykF | up | 2.013 | 0.003 |
| TYC81756.1 | 23S rRNA (cytosine(1962)-C(5))-methyltransferase RlmI | up | 1.958 | 0.032 |
| TYC82033.1 | glutamine--tRNA ligase | up | 1.887 | 0.023 |
| TYC79113.1 | tryptophan--tRNA ligase | up | 1.836 | 0.016 |
| TYC81811.1 | 3-phosphoserine/phosphohydroxythreonine transaminase | down | -1.527 | 0.021 |
| TYC80433.1 | aldo/keto reductase | down | -1.527 | 0.016 |
| TYC83257.1 | fumarate hydratase | down | -1.555 | 0.001 |
| TYC80545.1 | class II fructose-bisphosphate aldolase | down | -1.570 | 0.028 |
| TYC81196.1 | nucleoid-associated protein | down | -1.599 | 0.021 |
| TYC72577.1 | uroporphyrinogen-III C-methyltransferase | down | -1.644 | 0.021 |
| TYC83313.1 | 2-hydroxyacid dehydrogenase | down | -1.644 | 0.034 |
| TYC82406.1 | alpha-amylase | down | -1.674 | 0.028 |
| TYC79685.1 | glutathione synthase | down | -1.674 | 0.047 |
| TYC82747.1 | AAA family ATPase | down | -1.674 | 0.047 |
| TYC80326.1 | multidrug efflux RND transporter permease | down | -1.690 | 0.003 |
| TYC75534.1 | FKBP-type peptidyl-prolyl cis-trans isomerase | down | -1.737 | 0.008 |
| TYC81246.1 | NADH-quinone oxidoreductase subunit C/D | down | -1.753 | 0.004 |
| TYC80327.1 | multidrug efflux RND transporter periplasmic adaptor | down | -1.753 | 0.013 |
| TYC81177.1 | mannonate dehydratase | down | -1.753 | 0.018 |
| TYC82878.1 | aminobutyraldehyde dehydrogenase | down | -1.753 | 0.018 |
| TYC75575.1 | phosphoenolpyruvate carboxykinase (ATP) | down | -1.770 | 0.014 |
| TYC82002.1 | succinate dehydrogenase iron-sulfur subunit | down | -1.803 | 0.001 |
| TYC79654.1 | amidase/synthase | down | -1.803 | 0.001 |
| TYC83203.1 | carboxypeptidase M32 | down | -1.819 | 0.029 |
| TYC81691.1 | phosphatase | down | -1.819 | 0.036 |
| TYC81359.1 | transketolase | down | -1.836 | 4.29E-06 |
| TYC81243.1 | NADH-quinone oxidoreductase subunit NuoG | down | -1.836 | 0.001 |
| TYC78583.1 | sugar kinase | down | -1.870 | 0.032 |
| TYC83482.1 | aspartate aminotransferase family protein | down | -1.870 | 0.036 |
| TYC81164.1 | GTP cyclohydrolase I FolE | down | -1.887 | 0.031 |
| TYC83037.1 | E3 ubiquitin--protein ligase | down | -1.887 | 0.016 |
| TYC81973.1 | 6-phosphogluconolactonase | down | -1.905 | 0.016 |
| TYC79286.1 | UDP-N-acetylmuramoyl-L-alanyl-D-glutamate--2,6-diaminopimelate ligase | down | -1.923 | 0.004 |
| TYC81078.1 | dehydrogenase | down | -1.940 | 0.000 |
| TYC79690.1 | methionine adenosyltransferase | down | -1.940 | 0.002 |
| TYC81178.1 | fructuronate reductase | down | -1.940 | 0.003 |
| TYC81357.1 | malate dehydrogenase | down | -1.958 | 1.61E-05 |
| TYC79201.1 | murein transglycosylase | down | -1.976 | 0.004 |
| TYC79015.1 | isovaleryl-CoA dehydrogenase | down | -1.976 | 0.016 |
| TYC81462.1 | aminopeptidase PepB | down | -1.995 | 0.016 |
| TYC82381.1 | AMP nucleosidase | down | -1.995 | 0.002 |
| TYC82617.1 | murein tripeptide/oligopeptide ABC transporter ATP binding protein OppF | down | -2.013 | 0.011 |
| TYC82074.1 | alkyl hydroperoxide reductase subunit F | down | -2.032 | 0.006 |
| TYC83156.1 | cobalamin-independent methionine synthase II | down | -2.051 | 0.011 |
| TYC81218.1 | anaerobic glycerol-3-phosphate dehydrogenase | down | -2.089 | 2E-05 |
| TYC72539.1 | Xaa-Pro dipeptidase | down | -2.089 | 0.001 |
| TYC82627.1 | 6-phosphofructokinase II | down | -2.108 | 0.001 |
| TYC82385.1 | oxidoreductase | down | -2.128 | 0.002 |
| TYC81840.1 | hydroxylamine reductase | down | -2.147 | 1.61E-06 |
| TYC81526.1 | methionine--tRNA ligase | down | -2.147 | 0.001 |
| TYC81841.1 | NADH oxidoreductase | down | -2.167 | 0.001 |
| TYC76006.1 | Si-specific NAD(P)(+) transhydrogenase | down | -2.187 | 0.004 |
| TYC79555.1 | glucuronate isomerase | down | -2.187 | 0.001 |
| TYC81848.1 | lipoprotein | down | -2.187 | 0.046 |
| TYC78272.1 | AsmA family protein | down | -2.228 | 0.007 |
| TYC82886.1 | ABC transporter substrate-binding protein | down | -2.249 | 0.032 |
| TYC79316.1 | pyruvate dehydrogenase complex | down | -2.269 | 5.81E-05 |
| TYC82941.1 | alcohol dehydrogenase AdhP | down | -2.290 | 0.001 |
| TYC80464.1 | oxygen-insensitive NAD(P)H nitroreductase | down | -2.290 | 0.001 |
| TYC81220.1 | anaerobic glycerol-3-phosphate dehydrogenase | down | -2.333 | 0.001 |
| TYC82801.1 | cyclopropane fatty acyl phospholipid synthase | down | -2.754 | 0.025 |
| TYC78925.1 | inositol 2-dehydrogenase | down | -2.754 | 0.049 |
| TYC79193.1 | phosphopentomutase | down | -2.805 | 0.024 |
| TYC76039.1 | superoxide dismutase [Mn] | down | -2.805 | 0.003 |
| TYC83494.1 | NAD(P)H-quinone oxidoreductase | down | -4.365 | 3.32E-05 |
| TYC79191.1 | deoxyribose-phosphate aldolase | down | -4.405 | 1.18E-05 |
| TYC83288.1 | O-methyltransferase | down | -4.405 | 0.009 |
| TYC80758.1 | assimilatory sulfite reductase hemoprotein subunit | down | -4.613 | 5.23E-06 |
| TYC81321.1 | cysteine synthase A | down | -4.613 | 3.94E-05 |
| TYC81843.1 | ubiquinone-dependent pyruvate dehydrogenase | down | -4.698 | 0.001 |
| TYC83038.1 | NADH:flavin oxidoreductase/NADH oxidase | down | -5.011 | 9.02E-08 |
| TYC81998.1 | succinate--CoA ligase subunit alpha | down | -5.248 | 7.34E-05 |
| TYC79192.1 | thymidine phosphorylase | down | -5.296 | 2.53E-05 |
| TYC81881.1 | glutathione ABC transporter GsiB | down | -5.546 | 6.06E-05 |
| TYC78932.1 | DUF853 domain-containing protein | down | -5.970 | 6.56E-05 |
| TYC79643.1 | alcohol dehydrogenase | down | -6.081 | 0.001 |
| TYC75400.1 | 2-hydroxyacid dehydrogenase | down | -6.081 | 0.009 |
| TYC81718.1 | NAD(P)H:quinone oxidoreductase | down | -6.486 | 0.001 |
| TYC80201.1 | porphobilinogen synthase | down | -6.792 | 0.028 |
| TYC80759.1 | phosphoadenosine phosphosulfate reductase | down | -7.046 | 0.001 |
| TYC75409.1 | alpha-ketoacid dehydrogenase subunit beta | down | -7.311 | 0.001 |
| TYC83271.1 | type I glyceraldehyde-3-phosphate dehydrogenase | down | -8.241 | 0.001 |
| TYC80763.1 | sulfate adenylyltransferase subunit CysN | down | -8.953 | 0.001 |
| TYC80920.1 | TIM barrel protein | down | -10.471 | 2.57E-07 |
| TYC75404.1 | phospho-2-dehydro-3-deoxyheptonate aldolase | down | -13.061 | 0.009 |
| TYC81201.1 | malate dehydrogenase (quinone) | down | -19.408 | 0.001 |
| TYC82812.1 | alkene reductase | down | -33.113 | 0.018 |

Table S10. Nitrogen removal efficiency without or with IAA in different combinations of species *Enterobacter* and *Klebsiella*.

| Strain combinations | The ability of IAA production (mg/L) | The existence of the Iaa pathway | The existence of the Iac pathway | The ability of nitrate removal (mg/L*h) | The ability of nitrate removal with the addition of IAA (mg/L*h) |
| --- | --- | --- | --- | --- | --- |
| *Enterobacter* sp. Z1^α^ | 42.5±2.1 | + | + | 1.7±0.21 | 2.1±0.09 |
| *Enterobacter* sp. C1^β^ | 18.7±1.1 | - | + | 0.56±0.09 | 0.59±0.11 |
| *Enterobacter* sp. C7^β^ | 22.9±2.0 | + | + | 0.93±0.05 | 1.5±0.16 |
| *Enterobacter* sp. C9^β^ | 3.34±0.5 | - | - | 0.14±0.01 | 0.17±0.02 |
| *Enterobacter* sp. C12^β^ | 13.8±1.9 | + | - | 0.88±0.09 | 1.7±0.11 |
| *Klebsiella* sp. Z2^α^ | 52.1±3.2 | + | + | 1.7±0.13 | 2.8±0.71 |
| *Klebsiella* sp. Y1^β^ | 30.1±1.4 | + | + | 1.2±0.06 | 1.9±0.17 |
| *Klebsiella* sp. Y2^β^ | 40.8±2.6 | - | + | 0.58±0.07 | 1.1±0.02 |
| *Klebsiella* sp. N3^β^ | 5.17±1.5 | - | - | 0.26±0.03 | 0.23±0.05 |
| *Klebsiella* sp. X3^β^ | 9.92±0.89 | + | - | 0.19±0.02 | 0.32±0.04 |
| *Enterobacter* sp. Z1+ *Klebsiella* sp. Z2 | 58.7±1.3 | + | + | 2.8±0.18 | 4.5±0.37 |
| *Enterobacter* sp. C1+ *Klebsiella* sp. Z2 | 30.7±1.4 | + | + | 1.3±0.17 | 2.1±0.12 |
| *Enterobacter* sp. C9+ *Klebsiella* sp. Z2 | 49.3±3.6 | + | + | 1.9±0.21 | 3.5±0.34 |
| *Enterobacter* sp. Z1+ *Klebsiella* sp. Y1 | 33.2±2.9 | + | + | 2.1±0.26 | 3.5±0.25 |
| *Enterobacter* sp. Z1+ *Klebsiella* sp. Y2 | 45.6±3.1 | + | + | 1.6±0.08 | 1.9±0.14 |
| Other two strains combinations | About 10-20 | / | / | / | / |

^α^The strains were used in this study.

^β^The strains were obtained from our lab.

^γ^The value represents the mean of three replicates.

**References**

1. He, T., Li, Z., Sun, Q., Xu, Y., Ye, Q., 2016. Heterotrophic nitrification and aerobic denitrification by *Pseudomonas tolaasii* Y-11 without nitrite accumulation during nitrogen conversion. Bioresour. Technol. 200, 493-499.
2. Padhi, S.K., Tripathy, S., Sen, R., Mahapatra, A.S., Mohanty, S., Maiti, N.K., 2013. Characterisation of heterotrophic nitrifying and aerobic denitrifying *Klebsiella pneumoniae* CF-S9 strain for bioremediation of wastewater. Int. Biodeterior. Biodegrad. 78, 67-73.
3. Zhang, Y., Xu, Z., Li, J., Liu, D., Yuan, Y., Chen, Z., Wang, G., 2019. [Cooperation between two strains of *Enterobacter* and *Klebsiella* in the simultaneous nitrogen removal and phosphate accumulation processes.](https://pubmed.ncbi.nlm.nih.gov/31357041/) Bioresour. Technol. 291, 121854.
